# Comparison of Second-Line Treatments for Patients with Platinum-Resistant Recurrent or Metastatic Head and Neck Squamous Cell Carcinoma: A Systematic Review and Bayesian Network Meta-Analysis

**DOI:** 10.3390/cancers14184472

**Published:** 2022-09-15

**Authors:** Yan He, Junsong Zeng, Zhigong Wei, Yan Huang, Lianlian Yang, Xiaolin Hu, Yonglin Su, Xingchen Peng

**Affiliations:** 1 Department of Biotherapy, Cancer Center, West China Hospital, Sichuan University, Chengdu 610017, China; 2 Department of Nursing, West China Hospital, Sichuan University, Chengdu 610017, China; 3 Department of Rehabilitation, West China Hospital, Sichuan University, Chengdu 610017, China

**Keywords:** head and neck squamous cell carcinoma, recurrent or metastatic, second-line treatments, overall survival, Bayesian network meta-analysis

## Abstract

**Simple Summary:**

Which second-line treatment is the optimal choice for patients with platinum-resistant recurrent or metastatic head and neck squamous cell carcinoma remains unclear. To inform clinical decisions, we performed a network meta-analysis and systematic review to assess the relative efficacy and safety of second-line treatments for patients with platinum-resistant recurrent or metastatic head and neck squamous cell carcinoma. By synthesizing all available controlled trial evidence, PD-1 inhibitors significantly improved the overall survival, objective response rate, and treatment tolerance compared to the standard of care (docetaxel, methotrexate, or cetuximab). Afatinib presented a better progression-free survival and objective response rate than the standard of care. Compared with afatinib, PD-1 inhibitors had a better overall survival but a worse progress-free survival.

**Abstract:**

Several new drugs and combination strategies can be used to treat patients with recurrent or metastatic head and neck squamous cell carcinoma in the second-line treatment. Questions regarding the relative efficacy and safety of any two of the multiple second-line treatment strategies have emerged. This study aims to compare second-line treatments for patients with platinum-resistant recurrent or metastatic head and neck squamous cell carcinoma. Medline, Embase, and the Cochrane Central Register of Controlled Trials were searched to identify relevant articles. Direct and indirect evidence in terms of the objective response rate (ORR), progression-free survival (PFS), overall survival (OS), and treatment-related adverse events grade ≥ 3 (grade ≥ 3 trAE) were analyzed in this Bayesian network meta-analysis. A total of twenty-three trials involving 5039 patients were included. These studies compared 20 different treatments, including the standard of care (SOC: docetaxel, methotrexate, or cetuximab), PD-1 inhibitors (nivolumab or pembrolizumab), durvalumab, tremelimumab, durvalumab + tremelimumab, palbociclib + SOC, tivantinib + SOC, sorafenib + SOC, EMD1201081 + SOC, vandetanib + SOC, PX-866 + SOC, 5-fluorouracil + SOC, cixutumumab + SOC, gefitinib + SOC, cabazitaxel, nolatrexed, duligotuzumab, zalutumumab, gefitinib, and afatinib. Among the currently available treatment options, compared to the standard of care (SOC: docetaxel, methotrexate, or cetuximab), the PD inhibitor significantly improved OS, ORR, and grade ≥ 3 trAE. Afatinib presented a better PFS and ORR than the SOC. Compared with afatinib, the PD-1 inhibitor had a better OS but a worse PFS. In conclusion, compared to the SOC, the PD-1 inhibitor significantly improved the OS, ORR, and grade ≥ 3 trAE. Afatinib presented a better PFS and ORR than the SOC. Compared with afatinib, the PD-1 inhibitor had a better OS but a worse PFS.

## 1. Introduction

Squamous cell carcinoma of the head and neck remains a challenging clinical problem, with more than 300,400 new cases and 145,400 deaths reported annually worldwide [1]. Patients with recurrent or metastatic squamous cell carcinoma of the head and neck (R/M HNSCC) have a poor prognosis, and the median overall survival (OS) is less than one year [2]. Platinum-based chemotherapy has been a standard of care for R/M HNSCC in the first-line treatment [3]. After patients progressed on first-line treatments, the standard of care for second-line treatments is monotherapy of methotrexate, docetaxel, or cetuximab, which has yielded a median OS of 3 to 6 months [4]. Based on the unsatisfactory prognosis of R/M HNSCC, several strategies have been investigated to prolong the survival in second-line treatment. In recent decades, the strategies of second-line treatment have evolved from chemotherapy alone to targeted therapies, immunotherapies, and combination strategies, such as gefitinib [5], EMD1201081 [6], cixutumumab [7], tivantinib [8], vandetanib [9], sorafenib [10], nivolumab [11], and pembrolizumab [12].

As these new drugs and combination strategies can be used to treat patients with R/M HNSCC, questions regarding the relative efficacy and safety of these second-line treatment strategies have emerged. A previous network meta-analysis demonstrated that the PD-1 inhibitor presented the best OS and the fewest treatment-related adverse events grade ≥ 3 (grade ≥ 3 trAE), which divided second-line treatments into seven models (SOC, single or double targeted therapy, targeted therapy combined with chemotherapy, single or double immune checkpoint inhibitor therapy, and single chemotherapy) [13]. These categories may lead to internal heterogeneity because the effectiveness of these pharmaceuticals is different. Meanwhile, this categorization will neglect the disparity of some detailed comparisons. Therefore, a network meta-analysis comparing any two detailed regimens is helpful to evaluate the efficacy and safety of second-line treatment options for patients with platinum-resistant R/M HNSCC. Furthermore, previous studies recommended PD-1 inhibitors or afatinib to treat patients with platinum-resistant R/M HNSCC in the second-line treatment based on the evidence compared with the SOC. Among the regimens that showed a better effectiveness than the SOC, there is a lack of evidence regarding which is the best with respect to the overall survival, progression-free survival, and objective response rate. To inform clinical decisions, we performed this network meta-analysis using randomized controlled trials to assess the relative efficacy and safety of second-line treatments for patients with platinum-resistant R/M HNSCC.

## 2. Materials & Methods

This study was performed according to the preferred reporting items for systematic reviews and meta-analyses (PRISMA) extension statements for network meta-analyses. The protocol was registered in the Prospective Register of Systematic Reviews (PROSPERO CRD42020177194).

### 2.1. Data Sources and Searches

We searched Medline, Embase, and the Cochrane Central Register of Controlled Trials via Ovid to identify relevant articles reporting randomized clinical trials in August 2021. Only published data were used in the analysis unless the provided information was considered adequate. The detailed search strategies are shown in Table S1.

### 2.2. Study Selection

We included eligible studies that met the following criteria: (1) trials that enrolled patients with R/M HNSCC, (2) trials that enrolled patients progressing within 6 months after platinum-based chemotherapy, and (3) trials that reported at least one of the following clinical outcome measures: (a) objective response rate (ORR) assessed by the Response Evaluation Criteria in Solid Tumors (RECIST), (b) progression-free survival (PFS), (c) OS, and (d) the grade ≥ 3 trAE, as graded by the National Cancer Institute Common Toxicity Criteria for Adverse Events (CTCAE). Studies that did not meet the inclusion criteria were excluded.

### 2.3. Data Extraction and Risk of Bias Assessment

The following data were extracted: study and patient characteristics, treatments, and outcomes. If the articles did not report the hazard ratios (HR) and 95% confidence intervals (95%CI), these values were estimated from survival curves by Engauge Digitizer 4.1 according to the methods described by Tierney [14]. Any available sources for studies were assessed (including clinical trial registrations, preliminary results, meeting abstracts, etc.), and we preferred to use the latest and most complete data for the analysis. The Cochrane handbook for systematic reviews of interventions was used to assess the risk of bias, which evaluated the following study features: (a) allocation sequence generation; (b) allocation concealment; (c) blinding of participants and personnel; (d) blinding of the outcome assessment; (e) incomplete outcome data; (f) selective outcome reporting. Two investigators performed the study selection (Y He and JS Zeng), data extraction (Y He and YL Su), and assessment of the risk of bias (Y He and ZG Wei) independently. If disagreements occurred, they were settled by a discussion with all authors. XC Peng and XL Hu performed a review of the search findings from the search query results across the different databases evaluated. XC Peng and Y He performed the revision in order to ensure accurate capture of all relevant results.

### 2.4. Data Synthesis and Statistical Analysis

This Bayesian network meta-analysis was performed to synthesize direct and indirect evidence in terms of the ORR, PFS, OS, and grade ≥ 3 trAE. The results are reported as risk ratios (RR) for binary outcomes (ORR and grade ≥ 3 trAE) and HR for survival outcomes (PFS and OS) with 95% credible intervals. Network plots were used to illustrate the geometries to clarify which treatments were compared directly or indirectly in different outcomes. Pairwise meta-analyses were performed using the frequentist method by Stata 12.0 software (Stata Corporation, College Station, TX, USA) for the head-to-head comparisons. A network meta-analysis was performed with Bayesian models using the Markov chain Monte Carlo (MCMC) simulation technique in the R language and environment (R software, version 4.0.3) based on Just Another Gibbs Sampler (JAGS, version 4.3.0). In each analysis, we used four different sets of initial values based on 100,000 iterations after a burn-in of 50,000 with a thinning interval of one. Convergence was evaluated by a visual inspection of the four chains and the Brooks–Gelman–Rubin diagnostic. When posterior distributions are close to being normally distributed, credible intervals can be interpreted as conventional confidence intervals. The deviance information criteria (DIC) value was used to assess the fitness of the model. A lower DIC value indicated better model performance, and the model with the lower DIC value was used for the analysis. We assessed the heterogeneity among the studies using the I^2^ statistic, and estimated I^2^ values of <25%, 25% to 50%, and ≥50% were considered to indicate low, moderate, and high heterogeneity, respectively. If the I^2^ statistic was low or moderate, a fixed-effects model was used for the analysis. Otherwise, a random-effects model was used (Table S2). Contour-enhanced funnel plot was conducted to distinguish publication bias from other types of bias of the asymmetry using Stata version 12.0. The ranking probabilities for all treatments of being at each possible rank for each intervention were conducted, and the cumulative ranking-curve and its surface (SUCRA) were summarized. Transitivity is a key assumption underlying network meta-analyses that refers to the exchangeability across studies to compare treatment A with treatment B via treatment C. Transitivity was evaluated by the description of the patients’ characteristics and treatments. To minimize potential bias, only trials in which the regimens could not be transitive were used for the qualitative analysis. To confirm the robustness of the results, sensitivity analyses of only phase III trials were performed. Furthermore, since varied SOC regimens may represent a potential inherent bias in the network meta-analysis, we performed a sensitivity analysis that only included one regimen of SOC using available data.

## 3. Results

### 3.1. Study Characteristics

**Figure 1:**
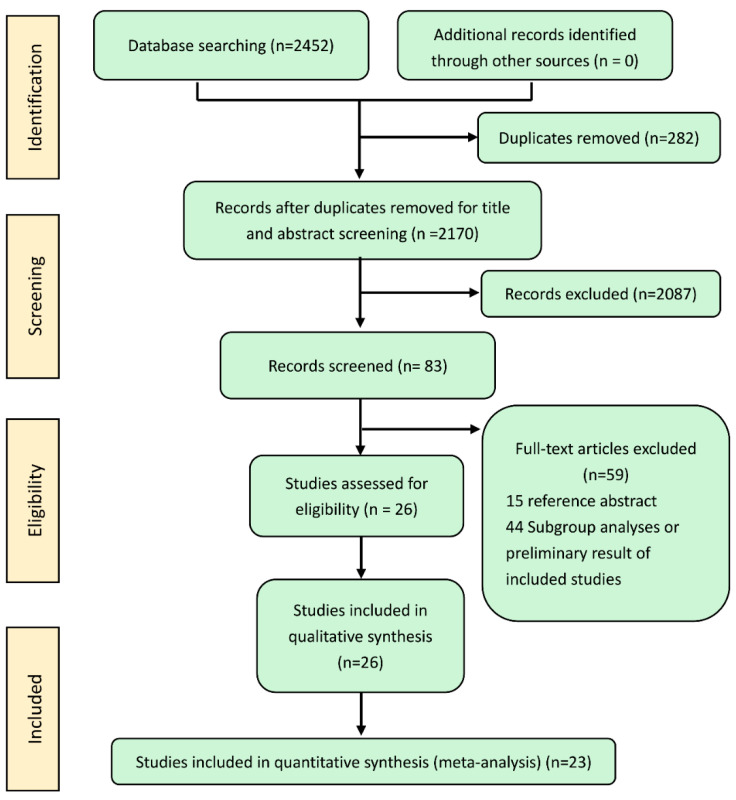
PRISMA flow diagram.

We identified 2452 records by searching the databases. After eliminating 282 duplicate records, 2087 records were removed based on the titles and abstracts, and the full texts of 83 records were reviewed. Finally, 26 randomized controlled trials were eligible for the qualitative synthesis. Three studies (the MAESTRO, E1304 and BERIL-1 study) [15,16,17] were excluded because their treatments (bortezomib + irinotecan versus bortezomib, Temsirolimus+ Cetuximab versus Temsirolimus, and buparlisib+ paclitaxel versus paclitaxel) could not validly compare two treatments via a connected indirect route. Finally, 23 trials involving 5039 patients [4,5,6,7,8,9,10,11,18,19,20,21,22,23,24,25,26,27,28,29,30,31,32] were included for the meta-analysis (Figure 1).

These studies described 20 different treatments, including the SOC (docetaxel, methotrexate, or cetuximab), PD-1 inhibitor (nivolumab or pembrolizumab), palbociclib + SOC, tivantinib + SOC, sorafenib + SOC, EMD1201081 + SOC, vandetanib + SOC, PX-866 + SOC, 5-fluorouracil (5-FU) +SOC, cixutumumab + SOC, gefitinib + SOC, cabazitaxel, nolatrexed, duligotuzumab, durvalumab, tremelimumab, durvalumab + tremelimumab, zalutumumab, gefitinib, and afatinib. Of these studies, a total of 8 studies were phase III clinical trials, and 13 studies were phase II, and another 2 studies did not definite the phase of the clinical trials. Only two studies were conducted in single-center, the others were conducted in a multicenter fashion. A total of 18 studies enrolled patients with a platinum-resistant disease, and 5 studies had no restrictions on prior platinum-based chemotherapy [5,19,24,28,30]. The median age of inclusion in the studies ranged from 42.5 to 63.6 years old. The ratio of male patients to female was approximately 5:1 in almost all of the included studies. The Eastern Cooperative Oncology Group performance status of all included patients was 0–2. Based on the available data, of the 2113 patients with an available tissue-reported HPV status, 608 patients (28.8%) were HPV-positive. The lowest proportion was reported from an Asian population (8.4%) [23]. The studies, which reported the EGFR status, observed that the proportion of EGFR expression-positive patients was more than 90% [5,22,24].

**Figure 2:**
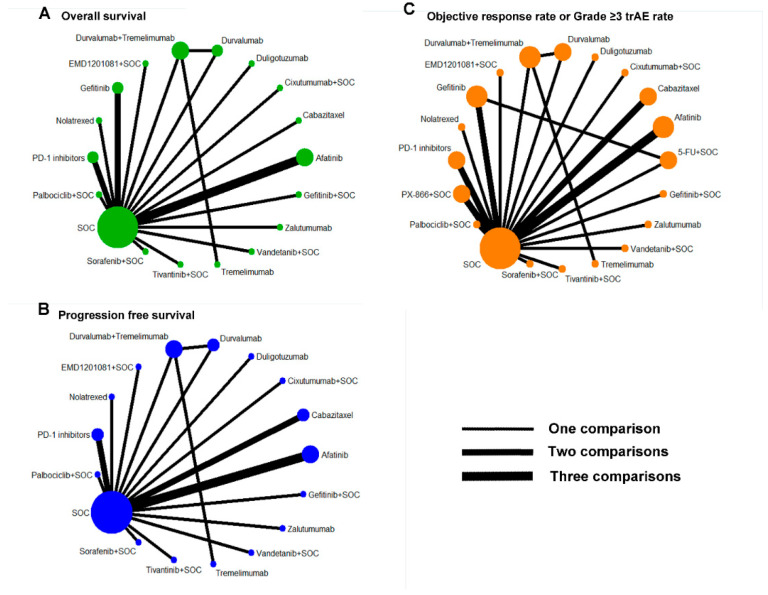
Network plot of (**A**) overall survival, (**B**) progression-free survival, (**C**) objective response rate, and grade ≥ 3 adverse events. SOC = standard of care (docetaxel, methotrexate, or cetuximab); 5-FU = 5-fluorouracil.

As shown in Table 1, when patients progressed after the first-line treatment, the ORR rates, median OS, and median PFS were 0–28.1%, 3.1–10.4 months, and 0.7–8.3 months. In detail, the highest ORR rates, median OS, and median PFS were reported by regimens of afatinib, buparlisib + paclitaxel, and the PD-1 inhibitor, respectively (Table 1). The network meta-analysis included 18 trials reporting the OS and 17 trials reporting the PFS, and 23 trials reporting the ORR and grade ≥ 3 trAE (Figure 2).

### 3.2. Quality Assessment

Almost all of these studies were high-quality and multicenter. In detail, as shown in Table S3, a total of 16, 16, 23, and 21 were evaluated as low risk with respect to their assessment for the method of randomization, allocation concealment, incomplete outcome data, and selective reporting. The number of studies with an unclear risk was seven in the method of randomization and seven in the allocation concealment, because the detailed method was unreported in these studies. We assessed two as “unclear risk” in selective reporting, because the PFS is an important outcome but they were not reported.

### 3.3. Network Meta-Analysis

**Figure 3:**
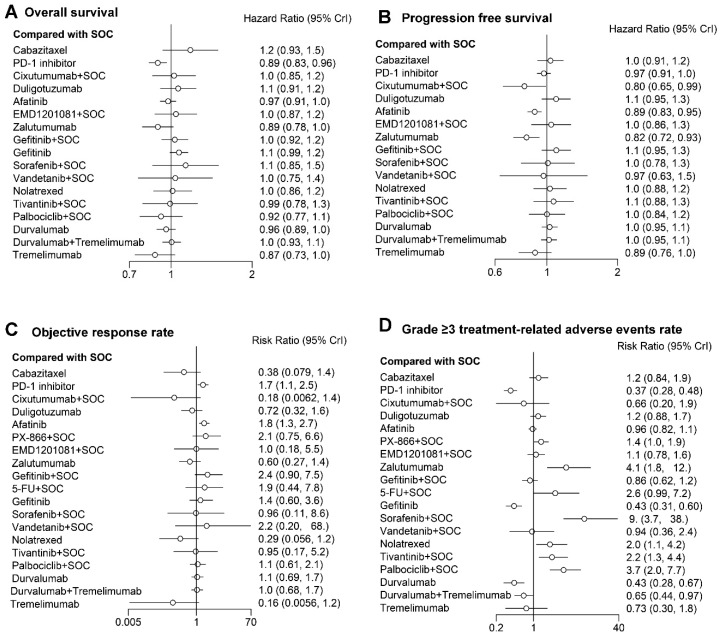
Forest plot of (**A**) overall survival (OS), (**B**) progression-free survival (PFS), (**C**) objective response rate (ORR), and (**D**) grade ≥ 3 treatment-related adverse events rates. SOC = standard of care (docetaxel, methotrexate, or cetuximab).

The PD-1 inhibitor may lead to improved trends in OS compared with the SOC (HR 0.89; 95% CI 0.83 to 0.96). Compared to the SOC, the addition of cixutumumab, EMD 1201081, gefitinib, vandetanib, sorafenib, tivantinib, and palbocilib failed to improve the OS. Regarding the PFS, among the EGFR monoclonal antibodies and TKIs, only afatinib significantly prolonged the PFS compared with the SOC (HR 0.89; 95% CI 0.83 to 0.95). In addition, the PFS of cixutumumab + SOC (HR 0.80; 95% CI 0.65 to 0.99) and zalutumumab (HR 0.82; 95% CI 0.72 to 0.93) were also significantly better than the SOC. Regarding the ORR, PD-1 inhibitors were similar to afatinib (RR 0.91; 95% CI 0.52 to 1.6). Meanwhile, among the second-line treatments for R/M HNSCC, only regimens of the PD-1 inhibitor (RR 1.7; 95% CI 1.1 to 2.5) and afatinib (RR 1.8; 95% CI 1.3 to 2.7) had a significantly higher ORR than the SOC. Furthermore, compared to the SOC, the addition of the PX-866, EMD 1201081, gefitinib, 5-Fu, vandetanib, sorafenib, tivantinib, and palbocilib failed to improve the ORR. For the grade ≥ 3 trAE, the frequencies of grade ≥ 3 trAE induced by the PD-1 inhibitor (RR 0.37; 95% CI 0.28 to 0.48), gefitinib (RR 0.43; 95% CI 0.31 to 0.6), durvalumab (RR 0.43; 95% CI 0.28 to 0.67), and durvalumab + tremelimumab (RR 0.65; 95% CI 0.44 to 0.97) were significantly lower than that induced by the SOC (Figure 3).

The PD-1 inhibitor and afatinib presented better OS, PFS, and ORR than the SOC. A new finding was that the OS of the PD-1 inhibitor was better than afatinib (HR 0.92; 95% CI 0.83 to 1.0), but the PFS was worse than afatinib (HR 1.1; 95% CI 1.0 to 1.2). Meanwhile, the ORR between the PD-1 inhibitor and afatinib was insignificant (RR 0.91; 95% CI 0.52 to 1.6), and the PD-1 inhibitor presented a significantly lower grade ≥ 3 trAE than afatinib (RR 0.39; 95% CI 0.28 to 0.53). We separated PD-L1 from PD-1 inhibitors and compared these two regimens because their mechanisms of action and treatment effectiveness were different. Our results showed that PD-1 inhibitors have a trend to prolong OSs compared to PD-L1 (PD-1 inhibitors versus durvalumab; HR 0.93; 95% CI, 0.83–1.0). The HR (PFS and OS) and RR (ORR and Grade ≥ 3 trAE) with a 95% CI between regimens of any two were shown in Figures S1 and S2.

### 3.4. Rank Probabilities

The rank probabilities of OS, PFS, ORR, and grade ≥ 3 trAE are shown in Figure S3. To identify the best treatment, the surface under the cumulative ranking curve (SUCRA) metric was used to rank the effectiveness and toxicity of each treatment. SUCRA values near one represent the best treatment with respect to the OS, PFS, and ORR. SUCRA values near to 0 represent the best treatment with respect to grade ≥ 3 trAE. In total, the PD-1 inhibitor (86%), tremelimumab (86%), and Zalutumumab (83%) had higher rankings in terms of OS. Regarding the PFS, zalutumumab (92%), cixutumumab + SOC (91%), and afatinib (81%) achieved high rankings among the other regimens. Gefitinib + SOC (84%), afatinib (79%) and PX-866 (79%) had a higher probability of providing a better ORR. The PD-1 inhibitor (4%), gefitinib (4%), and durvalumab (9%) had the probability of becoming the regimen with a lower frequency of grade ≥ 3 trAE. Zalutumumab showed a higher ranking in terms of OS and PFS, but the frequency of grade ≥ 3 trAE was also high (91%) (Table 2). However, SUCRA findings can be misleading and should be interpreted with caution. SUCRA does not show whether the difference between treatments is clinically meaningful. While one treatment may be rated as the best, the absolute difference between the best treatment and others may be trivial.

### 3.5. Heterogeneity and Inconsistency Assessment

The feasible pairwise comparisons with heterogeneity estimates are shown in Figures S4–S7. We observed consistency between the pairwise meta-analyses (direct evidence) and network meta-analyses (indirect evidence). Compared with the SOC, the PD-1 inhibitor significantly improved the OS (HR 0.77; 95% CI 0.65 to 0.91), and afatinib significantly prolonged the PFS (HR 0.76; 95% CI 0.65 to 0.88). Significantly higher ORRs than the SOC were obtained for the PD-1 inhibitor (RR 0.59; 95% CI 0.48 to 0.78) and gefitinib (RR 0.44; 95% CI 0.28 to 0.67). The PD-1 inhibitor showed a significantly lower frequency of grade ≥ 3 trAE than the SOC.

### 3.6. Sensitivity Analysis and Publication Bias

**Figure 4:**
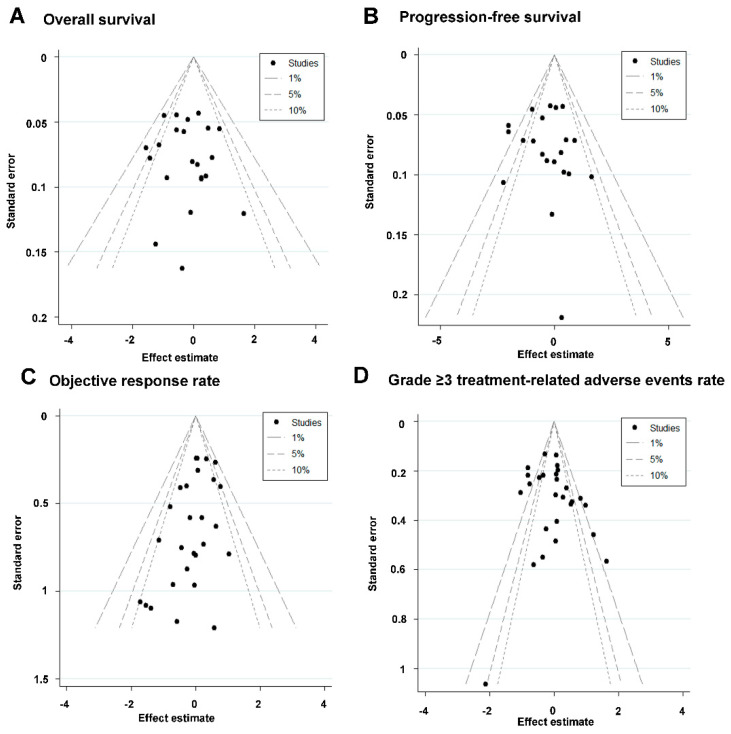
Contour-enhanced funnel plot of (**A**) overall survival (OS), (**B**) progression-free survival (PFS), (**C**) objective response rate (ORR), and (**D**) grade ≥ 3 treatment-related adverse event rates. SOC = standard of care (docetaxel, methotrexate, or cetuximab).

Considering the high quality of multinational explorative research and strict quality control, we conducted a sensitivity analysis only of phase III trials. Compared with the results of the overall network meta-analysis, the results of the sensitivity analysis in the phase III trials did not find relevant deviations (Figure S8). The results of the sensitivity analysis that only included one regimen of the SOC (docetaxel, methotrexate, or cetuximab) were consistent with the total analysis (Figures S9–S11). Contour-enhanced funnel plots were created to distinguish between publication bias and other causes of asymmetry. The contour-enhanced funnel plots were nearly symmetric, suggesting that there was no obvious publication bias (Figure 4).

## 4. Discussion

In this network meta-analysis, we have provided an overview of second-line regimens for patients with platinum-resistant R/M HNSCC. In summary, the PD-1 inhibitor significantly improved the OS and ORR, and afatinib significantly improved the PFS and ORR. Compared to the SOC, the addition of the PX-866, EMD 1201081, gefitinib, 5-Fu, vandetanib, sorafenib, tivantinib, and palbocilib failed to significantly improve the OS, PFS, or ORR. For the grade ≥ 3 trAE, the frequencies induced by the PD-1 inhibitor, gefitinib, and durvalumab were significantly lower than that induced by SOC.

The treatment for patients with R/M HNSCC after progressing from first-line treatment remains a challenging clinical problem [33]. From 2001 to 2020, although the OS of patients with R/M HNSCC after progressing from first-line treatment improved from 3.1 months to 7–9 months, the treatment effectiveness remains far from satisfactory [8,19]. During the past two decades, researchers have conducted numerous explorations, such as targeted therapies, immunotherapies, and novel combination strategies [34]. However, most therapeutic strategies fail to improve the effectiveness [27,30]. In our analysis, among 20 different regimens, a total of 17 regimens could not prolong the OS or PFS, and 5 regimens (zalutumumab, sorafenib + SOC, vandetanib + SOC, tivantinib + SOC, and nolatrexed) presented a higher frequency of grade ≥ 3 trAE. Although another three regimens (the PD-1 inhibitor, afatinib, and zalutumumab) significantly improved survival, the prolonging of the overall survival still remained limited. For example, regarding the PD-1 inhibitor versus the SOC, the median OS was 8.4 versus 6.9 months in the KEYNOTE-040 trial [4] and 7.5 versus 5.1 months in the CheckMate 141 trial [11]. Therefore, based on the unsatisfactory prognosis in the second-line treatment for R/M HNSCC, new drugs, novel combination strategies, and divided patients according to biomarkers for a precision approach are warranted.

HNSCC patients presented with different subgroups of interest in relation to the potential impact on the therapeutic management, including the status of PD-L1, HPV, and EGFR. It was reported that HPV-positive HNSCC showed a poor response to many drugs, such as cetuximab [8,27], afatinib [20], duligotuzumab [27], tivantinib [8], and methotrexate [20]. Specifically, a study reported that the response rate in 31 HPV-positive patients was 0% in both arms (tivantinib + cetuximab and cetuximab), which was consistent with that reported of another three drugs (afatinib, duligotuzumab, and methotrexate) [8,20,27]. However, the PD-1 inhibitor showed promising effectiveness in HPV-positive patients. Compared with the SOC, the PD-1 inhibitor significantly improved the OS, and the median overall survival was 9.1 months for the PD-1 inhibitor versus 4.4 months in the SOC (HR 0.56; 95% CI 0.32 to 0.99) [11]. The status of EGFR was found to be positive in more than 90% of HNSCC patients, so it was not correlated with survival and might be a promising therapeutic target [24]. The drugs used for second-line treatments of R/M HNSCC targeting the EGFR pathway included anti-EGFR monoclonal antibodies (zalutumumab, cetuximab, vandetanib, and duligotuzumab) and EGFR-TKIs (afatinib and gefitinib) [22,27,35]. Previous studies reported that targeting the EGFR signaling pathway presented promising antitumor activity in R/M HNSCC [4,11,36]. However, we found that among new attempts to target EGFR, only afatinib showed promising effectiveness in improving the ORR and PFS compared with SOC. Adding a new drug to the SOC was an important way to improve the survival outcome, but we found that only cixutumumab + SOC significantly prolonged PFS. Most of the combination regimens did not cover the SOC alone, such as PX-866 + SOC, EMD1201081 + SOC, sorafenib + SOC, tivantinib + SOC, or palbocilib + SOC. New chemotherapy drugs, such as cabazitaxel and nolatrexed, failed to improve the effectiveness of the second-line treatment in patients with R/M HNSCC.

In consideration of afatinib and the PD-1 inhibitor showing effective antitumor activity, future trials need to highlight head-to-head comparisons of the PD-1 inhibitor versus afatinib. It is also worth confirming the effectiveness of some new combination regimens, such as afatinib + SOC, afatinib + PD-1 inhibitor, and SOC + PD-1 inhibitor in second-line treatments. We also found that there are several ongoing trials evaluating the effectiveness and toxicity of second-line treatments, such as nab-paclitaxel + nivolumab (NCT04831320), tazemetostat + pembrolizumab (NCT04831320), INCAGN01876 + retifanlimab (NCT05359692), MRG003, monalizumab + cetuximab (NCT04590963), lenvatinib + pembrolizumab (NCT04428151), pembrolizumab + cetuximab (NCT03082534), docetaxel + nivolumab (NCT05027204), NC-6004 + pembrolizumab (NCT03771820), duvelisib + docetaxel (NCT05057247), and paclitaxel + cetuximab (NCT04278092).

This study has several advantages. Previous meta-analyses were usually focused on the effectiveness of immune checkpoint inhibitors, and they supported our results that immune checkpoint inhibitors can improve the treatment effectiveness [37,38,39,40]. In our study, we were not only concerned about immune checkpoint inhibitors but also paid attention to other regimens. Another meta-analysis was performed to compare multiple treatments but only pairwise comparisons were used [41]. A previous network meta-analysis compared seven categories in second-line treatments, including the SOC, single or double targeted therapy, targeted therapy combined with chemotherapy, single or double immune checkpoint inhibitor therapy, and single chemotherapy [13]. In our network meta-analysis, the comparisons of any of two detailed regimens were helpful for evaluating the efficacy and safety of second-line treatment options. Furthermore, the pairwise analysis and the sensitivity analysis were all consistent with the results from the initial network meta-analysis. This proved that the results of our analysis were more robust. Overall, by synthesizing all available controlled trial evidence, this network meta-analysis comprehensively reported the second-line treatments of patients with platinum-resistant R/M HNSCC to provide a reference source for evaluating the strengths and weaknesses with respect to treatment selection among multiple options in clinical practice.

This study also had some limitations. First, there were some unavoidable confounding factors because the comparison of treatments was indirect and most of the direct evidence was from one trial. Second, because phase II clinical trials usually have small sample sizes with inadequate statistical power, the results should be interpreted with caution. Third, given the small number of trials reporting the level of EGFR or PD-1 expression, we did not perform a subgroup analysis to assess the efficacy and safety of these treatments in patients with different EGFR or PD-1 expression levels. Finally, among the included studies, we attempted to obtain a comparison using one regimen of the SOC (docetaxel, methotrexate, or cetuximab) in the total analysis. Unfortunately, several crucial clinical trials could not report the separate results. Varied SOC regimens might present a potential bias. In addition, due to our results mainly originating from platinum-resistant patients, some results might not extend to patients who progress from other regimens.

## 5. Conclusions

Among the currently available treatment options, compared to the SOC, PD-1 inhibitors significantly improved overall survival, objective response rate, and treatment tolerance. Afatinib presented a better progression-free survival and objective response rate than the SOC. Compared with afatinib, the PD-1 inhibitor had a better overall survival but a worse progression-free survival. Compared to the SOC, the addition of the PX-866, EMD1201081, gefitinib, 5-fluorouracil, vandetanib, sorafenib, tivantinib, and palbocilib failed to significantly improve the overall survival, progression-free survival, or objective response rate.

## Figures and Tables

**Table 1:** Characteristics of the included studies.

Trials	Phase	Multi- Center	Inclusion Period	Treatments	Number of Patients	Median Age (Years)	Median OS (Months)	Median PFS (Months)	ORR rates (%)	Grade ≥ 3 trAE Rates (%)
Kochanny et al., 2020 [8]	II	yes	2012 to 2014	Tivantinib + SOC SOC	40 38	60.5 63.6	7.4 8.6	3.5 3.5	7.5 7.9	67.50 26.32
Douglas et al., 2021 [29]	II	yes	2015 to 2017	Palbociclib + SOC SOC	65 60	NA NA	9.7 7.8	3.9 4.6	27.7 25	53.13 15.00
Guo et al., 2019 [23]	III	yes	2013 to 2018	Afatinib SOC	228 112	55.5 58	6.9 6.4	2.9 2.6	28.1 13.4	39.04 59.82
Cohen et al., 2019 [4]	III	yes	2014 to 2016	PD-1 inhibitors SOC	247 248	60 60	8.4 6.9	8.3 6.6	14.6 10.1	13.41 36.32
Ferrarotto et al., 2018 [7]	II	yes	2008 to 2010	Cixutumumab + SOC SOC	47 44	NA NA	5.3 5.5	1.9 2.0	2.1 9.1	10.64 15.91
Joshi et al., 2017 [30]	II	no	2015 to 2016	Cabazitaxel SOC	46 46	47.5 42.5	3.833 5.16	0.7 2.03	2.2 13.6	36.96 34.78
Machiels et al., 2016 [21]	II	yes	2012 to 2014	Cabazitaxel SOC	53 48	58 57.5	5 3.6	1.9 1.9	0 2.1	39.62 27.08
Ferris et al., 2016 [11]	III	yes	2014 to 2015	PD-1 inhibitor SOC	240 121	59 61	7.5 5.1	2.0 2.3	13.3 5.8	13.14 35.14
Fayette et al., 2016 [27]	II	yes	2012 to 2013	Duligotuzumab SOC	59 62	62 62	7.2 8.7	4.2 4	15.3 21.0	61.02 50.00
Machiels et al., 2015 [20]	III	yes	2012 to 2013	Afatinib SOC	322 161	60 59	6.8 6.0	2.6 1.7	10.2 5.6	39.69 35.63
Jimeno et al., 2015 [25]	II	yes	2012 to 2013	PX-866 + SOC SOC	42 41	59 63	7.03 8.53	2.67 2.67	9.5 7.3	62.50 53.85
Jimeno et al., 2015 [26]	II	yes	2011 to 2013	PX-866 + SOC SOC	42 43	62 60	8.76 6.5	3.07 2.73	14.3 4.7	61.90 34.88
Gilbert et al., 2015 [10]	II	yes	2009 to 2011	SOC Sorafenib + SOC	27 28	59 60	9 5.7	3 3.2	7.4 7.1	11.11 92.86
Seiwert et al., 2014 [18]	II	yes	2007 to 2011	Afatinib SOC	62 62	58 58	9 11.8	3.25 3.5	8.1 9.7	51.61 17.74
Ruzsa et al., 2014 [6]	II	yes	2009 to 2012	EMD1201081 + SOC SOC	53 53	58 57	6.3 NA	1.5 1.9	5.7 5.7	56.60 50.94
Limaye et al., 2013 [9]	II	yes	2007 to 2009	SOC Vandetanib + SOC	14 15	56 60	6.7 6.03	0.8 2.25	7.1 13.3	42.86 40.00
Argiris et al., 2013 [28]	III	yes	2004 to 2008	SOC Gefitinib + SOC	117 122	60.8 61.4	6.0 7.3	2.1 3.5	4.3 9.8	41.03 35.25
Machiels et al., 2011 [22]	III	yes	2006 to 2009	Zalutumumab SOC	191 95	57 58	6.7 5.2	2.48 2.1	6.3 10.5	20.63 5.32
Pivot et al., 2001 [19]	NA	yes	1997 to 1998	Nolatrexed SOC	93 46	57.9 62	3.1 3.1	1.9 1.5	3.2 10.9	38.71 19.57
Kushwaha et al., 2015 [24]	NA	no	2010 to 2012	Gefitinib SOC 5-Fu + SOC	39 40 38	NA NA NA	8.8 7.8 8.1	NA NA NA	7.7 5.0 7.9	2.56 12.50 26.32
Stewart et al., 2009 [5]	III	yes	2003 to 2006	Gefitinib SOC	325 161	NA NA	6 6.7	NA NA	2.5–7.6 3.9	10.13–19.88 35.22
Siu et al., 2019 [31]	II	yes	2015 to 2016	Durv Treme Durv + Treme	67 67 133	62 61 62	6.0 5.5 7.6	1.9 1.9 2.0	9.2 1.6 7.8	12.31 16.92 15.79
Ferris et al., 2020 [32]	III	yes	2015 to 2017	Durv Durv + Treme SOC	240 247 249	60	7.6 6.5 8.3	2.1 2.0 3.7	17.9 18.2 17.3	10.1 16.3 24.2

Abbreviations: OS = overall survival, PFS = progression-free survival, ORR = objective response rate, Grade ≥ 3 trAE = grade ≥ 3 treatment-related adverse event rates, NA = not available, SOC = standard of care (docetaxel, methotrexate or cetuximab), 5-FU = 5-fluorouracil, Durva = Durvalumab, and Treme = Tremelimumab.

**Table 2:** The summarized SUCRA (ranks) with respect to the effectiveness and toxicity of each treatment. SUCRA values near to 1 entail the best treatment with respect to OS, PFS, and OR. SUCRA values near to 0 entail the best treatment with respect to grade ≥ 3 trAE. SOC = standard of care (docetaxel, methotrexate, or cetuximab). The darker the color represented higher probability.

Sucra (Ranks)	OS	PFS	ORR Rates	Grade ≥ 3 trAE Rates
SOC	49%	48%	47%	43%
Cabazitaxel	14%	36%	19%	57%
PD-1 inhibitor	86%	60%	75%	4%
Cixutumumab + SOC	42%	91%	11%	27%
Duligotuzumab	31%	20%	34%	56%
Afatinib	60%	81%	79%	40%
PX-866 + SOC	NA	NA	79%	64%
EMD1201081 + SOC	37%	35%	51%	50%
Zalutumumab	83%	92%	29%	91%
Gefitinib + SOC	38%	20%	84%	33%
Vandetanib + SOC	42%	53%	72%	41%
Nolatrexed	45%	38%	15%	76%
5-FU + SOC	NA	NA	73%	80%
Gefitinib	25%	NA	64%	9%
Sorafenib + SOC	23%	45%	50%	99%
Tivantinib + SOC	52%	30%	49%	79%
Palbociclib + SOC	74%	47%	54%	89%
Durvalumab	66%	37%	52%	9%
Durva + Treme	47%	40%	51%	22%
Tremelimumab	86%	78%	10%	30%

## Data Availability

Datasets are available on reasonable request from the corresponding author.

## Supplementary Materials

The following supporting information can be downloaded at: https://www.mdpi.com/article/10.3390/cancers14184472/s1, Table S1: Search strategy via Ovid; Table S2: Heterogeneity test and DIC values; Table S3: Summary of the risk of bias assessment; Figure S1: Network analysis of overall survival and progression free survival; Figure S2: Network analysis of objective response rate and treatment-related adverse events grade ≥ 3 adverse events; Figure S3: Rank probabilities; Figure S4: Pairwise meta-analyses in overall survival; Figure S5: Pairwise meta-analyses in progression free survival; Figure S6: Pairwise meta-analyses in objective response rate; Figure S7: Pairwise meta-analyses in adverse events of grade 3 or higher; Figure S8: Sensitivity analysis in the phase III trials; Figure S9: Sensitivity analysis of methotrexate; Figure S10: Sensitivity analysis of cetuximab; Figure S11: Sensitivity analysis of docetaxel.
