# Supplementary material for: Comparison of Second-Line Treatments for Patients with Platinum-Resistant Recurrent or Metastatic Head and Neck Squamous Cell Carcinoma: A Systematic Review and Bayesian Network Meta-Analysis
Source: Cancers (Basel). 2022 Sep 15;14(18):4472. doi: 10.3390/cancers14184472 (PMC9497217; doi:10.3390/cancers14184472)

Supplementary Material

Table S1. Search strategy via Ovid........2
Table S2. Heterogeneity test and DIC values........3
Table S3. Summary of the risk of bias assessment........3
Figure S1. Network analysis of overall survival and progression free survival........4
Figure S2. Network analysis of objective response rate and treatment-related adverse events grade ≥3 adverse events ...5
Figure S3. Rank probabilities........6
Figure S4. Pairwise meta-analyses in overall survival........8
Figure S5. Pairwise meta-analyses in progression free survival........8
Figure S6. Pairwise meta-analyses in objective response rate........9
Figure S7. Pairwise meta-analyses in adverse events of grade 3 or higher........10
Figure S8. Sensitivity analysis in the phase III trials.........11
Figure S9. Sensitivity analysis of methotrexate........11
Figure S10. Sensitivity analysis of cetuximab........12
Figure S11. Sensitivity analysis of docetaxel........12

Table S1. Search strategy via Ovid.

Embase 1974 to 2021 August 20; Ovid MEDLINE(R) 1946 to August Week 2 2021; EBM Reviews - Cochrane Central Register of Controlled Trials July 2021.

1	exp Neoplasms/
2	(Neoplasia or Neoplasm or Tumor or Cancer or Malignancy or Carcinoma or Neoplasias or Neoplasms or Tumors or Cancers or Malignancies or Carcinomas). ab,ti.
3	1 or 2
4	(Head or neck or oral cavity or pharynx or larynx or Tongue or Mouth or Otorhinolaryngology or oropharynx). ab,ti.
5	(Recurrent or metastatic).ab,ti.
6	3 and 4 and 5
7	exp Drug Therapy/
8	exp Immunotherapy/
9	(chemotherapy or chemotherapies or cetuximab or docetaxel or methotrexate or paclitaxel or platinum or PD-1 or PD-L1 or EGFR or epidermal growth factor receptor).ab,ti.
10	7 or 8 or 9
11	6 and 10
12	randomized controlled trial.pt.
13	controlled clinical trial.pt.
14	random*.mp.
15	placebo.ab.
16	drug therapy.fs.
17	trial.ab.
18	groups.ab.
19	12 or 13 or 14 or 15 or 16 or 17 or 18
20	exp animals/ not humans.sh.
21	19 not 20
22	6 and 10 and 21
23	limit 22 to (yr="2000 - 2021" and english language)

Table S2. Heterogeneity test and DIC values.

	I^2	DIC value	Models
OS	0%	35.7	Fixed-effects model
PFS	12%	38.7	Fixed-effects model
ORR	4%	102.9	Fixed-effects model
AE	36%	129.1	Fixed-effects model

Table S3. Summary of the risk of bias assessment.

Trials	*Method of randomization*	*Allocation concealment*	*blinded*	*Incomplete outcome data*	*Selective reporting*
Kochanny et al. 2020	Low risk	Low risk	Open-label	Low risk	Low risk
Douglas et al. 2021	Low risk	Low risk	Double-blind	Low risk	Low risk
Guo et al. 2019	Low risk	Low risk	Open-label	Low risk	Low risk
Cohen et al. 2019	Low risk	Low risk	Open-label	Low risk	Low risk
Ferrarotto et al. 2018	Unclear risk	Unclear risk	Open-label	Low risk	Low risk
Joshi et al. 2017	Low risk	Low risk	Open-label	Low risk	Low risk
Machiels et al. 2016	Unclear risk	Unclear risk	Open-label	Low risk	Low risk
Ferris et al. 2016	Low risk	Low risk	Open-label	Low risk	Low risk
Fayette et al. 2016	Low risk	Low risk	Open-label	Low risk	Low risk
Machiels et al. 2015	Low risk	Low risk	Open-label	Low risk	Low risk
Jimeno et al. 2015	Low risk	Low risk	Open-label	Low risk	Low risk
Jimeno et al. 2015	Low risk	Low risk	Open-label	Low risk	Low risk
Gilbert et al. 2015	Low risk	Low risk	Double-blind	Low risk	Low risk
Seiwert et al. 2014	Low risk	Low risk	Open-label	Low risk	Low risk
Ruzsa et al. 2014	Unclear risk	Unclear risk	Open-label	Low risk	Low risk
Limaye et al. 2013	Unclear risk	Unclear risk	Open-label	Low risk	Low risk
Argiris et al. 2013	Low risk	Low risk	Double-blind	Low risk	Low risk
Machiels et al. 2011	Low risk	Low risk	Open-label	Low risk	Low risk
Pivot et al. 2001	Unclear risk	Unclear risk	Open-label	Low risk	Low risk
Kushwaha et al. 2015	Unclear risk	Unclear risk	Open-label	Low risk	Unclear risk
Stewart et al. 2009	Unclear risk	Unclear risk	Double-blind	Low risk	Unclear risk
Siu et al. 2019	Low risk	Low risk	Open-label	Low risk	Low risk
Ferris et al. 2020	Low risk	Low risk	Open-label	Low risk	Low risk

Figure S1. Network analysis of overall survival and progression free survival.

Progression free survival (HR, 95%CI) →

Overall survival (HR. 95%CI) ↓

**SOC**	0.97 (0.85, 1.1)	1 (0.97, 1.1)	1.2 (1.0, 1.5)	0.91 (0.79, 1.1)	1.1 (1.1, 1.2)	0.96 (0.79, 1.2)	1.2 (1.1, 1.4)	0.91 (0.79, 1.1)		0.99 (0.76, 1.3)	1 (0.67, 1.6)	0.97 (0.83, 1.1)	0.94 (0.77, 1.1)	1 (0.84, 1.2)	0.97 (0.90, 1.1)	0.98 (0.91, 1.1)	1.1 (0.96, 1.3)
0.85 (0.67, 1.1)	**Cabazitaxel**	1.1 (0.93, 1.2)	1.3 (1.0, 1.7)	0.95 (0.78, 1.1)	1.2 (1.0, 1.3)	0.99 (0.79, 1.2)	1.3 (1.1, 1.5)	0.94 (0.78, 1.1)		1 (0.77, 1.4)	1.1 (0.68, 1.7)	1 (0.82, 1.2)	0.97 (0.77, 1.2)	1 (0.83, 1.3)	1 (0.87, 1.2)	1 (0.88, 1.2)	1.2 (0.95, 1.4)
1.1 (1.0, 1.2)	1.3 (1.0, 1.7)	**PD-1 inhibitor**	1.2 (0.97, 1.5)	0.89 (0.76, 1.0)	1.1 (1.0, 1.2)	0.93 (0.76, 1.1)	1.2 (1.0, 1.4)	0.89 (0.76, 1.0)		0.96 (0.74, 1.3)	1 (0.65, 1.5)	0.94 (0.79, 1.1)	0.91 (0.74, 1.1)	0.97 (0.80, 1.2)	0.94 (0.85, 1.0)	0.95 (0.86, 1.1)	1.1 (0.92, 1.3)
0.97 (0.81, 1.2)	1.1 (0.85, 1.6)	0.87 (0.71, 1.1)	**Cixutumumab+SOC**	0.73 (0.57,0.94)	0.9 (0.73, 1.1)	0.77 (0.58, 1.0)	0.98 (0.77, 1.3)	0.73 (0.57,0.94)		0.79 (0.57, 1.1)	0.83 (0.51, 1.3)	0.78 (0.60, 1.0)	0.75 (0.56, 1.0)	0.8 (0.61, 1.1)	0.78 (0.62,0.97)	0.79 (0.63,0.98)	0.9 (0.69, 1.2)
0.94 (0.81, 1.1)	1.1 (0.84, 1.5)	0.84 (0.71,0.99)	0.97 (0.76, 1.2)	**Duligotuzumab**	1.2 (1.1, 1.4)	1 (0.83, 1.3)	1.3 (1.1, 1.6)	1 (0.82, 1.2)		1.1 (0.81, 1.5)	1.1 (0.72, 1.8)	1.1 (0.86, 1.3)	1 (0.81, 1.3)	1.1 (0.87, 1.4)	1.1 (0.91, 1.3)	1.1 (0.91, 1.3)	1.2 (0.99, 1.5)
1 (0.96, 1.1)	1.2 (0.95, 1.5)	0.92 (0.83, 1.0)	1.1 (0.87, 1.3)	1.1 (0.92, 1.3)	**Afatinib**	0.85 (0.69, 1.0)	1.1 (0.94, 1.2)	0.81 (0.69,0.94)		0.88 (0.67, 1.2)	0.92 (0.59, 1.4)	0.86 (0.72, 1.0)	0.83 (0.68, 1.0)	0.89 (0.73, 1.1)	0.86 (0.78,0.95)	0.87 (0.79,0.96)	1 (0.84, 1.2)
0.96 (0.80, 1.1)	1.1 (0.84, 1.5)	0.86 (0.71, 1.0)	0.98 (0.76, 1.3)	1 (0.81, 1.3)	0.94 (0.77, 1.1)	**EMD1201081+SOC**	1.3 (1.0, 1.6)	0.95 (0.75, 1.2)		1 (0.75, 1.4)	1.1 (0.67, 1.7)	1 (0.79, 1.3)	0.98 (0.74, 1.3)	1 (0.80, 1.4)	1 (0.83, 1.2)	1 (0.83, 1.3)	1.2 (0.91, 1.5)
1.1 (0.98, 1.3)	1.3 (1.0, 1.7)	1 (0.86, 1.2)	1.1 (0.92, 1.4)	1.2 (0.97, 1.5)	1.1 (0.94, 1.3)	1.2 (0.93, 1.5)	**Zalutumumab**	0.75 (0.62,0.90)		0.81 (0.61, 1.1)	0.85 (0.54, 1.3)	0.79 (0.65, 0.97)	0.77 (0.61,0.97)	0.82 (0.66, 1.0)	0.8 (0.69, 0.92)	0.8 (0.69, 0.93)	0.92 (0.75, 1.1)
0.97 (0.87, 1.1)	1.1 (0.88, 1.5)	0.86 (0.75,0.99)	0.99 (0.80, 1.2)	1 (0.85, 1.2)	0.94 (0.83, 1.1)	1 (0.82, 1.2)	0.87 (0.73, 1.0)	**Gefitinib+SOC**		1.1 (0.81, 1.5)	1.1 (0.72, 1.8)	1.1 (0.86, 1.3)	1 (0.81, 1.3)	1.1 (0.87, 1.4)	1.1 (0.91, 1.3)	1.1 (0.92, 1.3)	1.2 (1.0, 1.5)
0.93 (0.87, 1.0)	1.1 (0.86, 1.4)	0.83 (0.75,0.93)	0.96 (0.79, 1.2)	0.99 (0.84, 1.2)	0.91 (0.82, 1.0)	0.97 (0.80, 1.2)	0.83 (0.72,0.97)	0.96 (0.84, 1.1)	**Gefitinib**								
0.88 (0.67, 1.2)	1 (0.72, 1.5)	0.79 (0.59, 1.1)	0.91 (0.65, 1.3)	0.94 (0.68, 1.3)	0.86 (0.64, 1.1)	0.92 (0.66, 1.3)	0.79 (0.58, 1.1)	0.91 (0.67, 1.2)	0.94 (0.71, 1.3)	**Sorafenib+SOC**	1 (0.63, 1.7)	0.98 (0.72, 1.3)	0.95 (0.68, 1.3)	1 (0.74, 1.4)	0.98 (0.75, 1.3)	0.99 (0.75, 1.3)	1.1 (0.84, 1.5)
0.96 (0.70, 1.3)	1.1 (0.76, 1.7)	0.86 (0.62, 1.2)	0.99 (0.69, 1.4)	1 (0.72, 1.5)	0.94 (0.68, 1.3)	1 (0.70, 1.4)	0.86 (0.61, 1.2)	1 (0.71, 1.4)	1 (0.74, 1.4)	1.1 (0.71, 1.7)	**Vandetanib+SOC**	0.94 (0.59, 1.5)	0.91 (0.57, 1.5)	0.97 (0.61, 1.5)	0.94 (0.61, 1.5)	0.95 (0.61, 1.5)	1.1 (0.69, 1.7)
0.99 (0.84, 1.2)	1.2 (0.87, 1.6)	0.88 (0.74, 1.1)	1 (0.79, 1.3)	1 (0.84, 1.3)	0.96 (0.81, 1.1)	1 (0.81, 1.3)	0.88 (0.71, 1.1)	1 (0.84, 1.2)	1.1 (0.88, 1.3)	1.1 (0.81, 1.5)	1 (0.72, 1.5)	**Nolatrexed**	0.97 (0.75, 1.2)	1 (0.81, 1.3)	1 (0.84, 1.2)	1 (0.85, 1.2)	1.2 (0.92, 1.5)
1 (0.80, 1.3)	1.2 (0.85, 1.7)	0.9 (0.70, 1.2)	1 (0.77, 1.4)	1.1 (0.81, 1.4)	0.98 (0.77, 1.3)	1.1 (0.78, 1.4)	0.9 (0.69, 1.2)	1 (0.80, 1.3)	1.1 (0.84, 1.4)	1.1 (0.79, 1.6)	1 (0.70, 1.6)	1 (0.77, 1.4)	**Tivantinib+SOC**	1.1 (0.82, 1.4)	1 (0.84, 1.3)	1 (0.85, 1.3)	1.2 (0.93, 1.5)
1.1 (0.91, 1.3)	1.3 (0.95, 1.7)	0.97 (0.80, 1.2)	1.1 (0.86, 1.4)	1.2 (0.91, 1.5)	1.1 (0.87, 1.3)	1.1 (0.88, 1.5)	0.97 (0.78, 1.2)	1.1 (0.91, 1.4)	1.2 (0.96, 1.4)	1.2 (0.88, 1.7)	1.1 (0.78, 1.6)	1.1 (0.86, 1.4)	1.1 (0.80, 1.5)	**Palbociclib+SOC**	0.97 (0.80, 1.2)	0.98 (0.81, 1.2)	1.1 (0.89, 1.4)
1 (0.96, 1.1)	1.2 (0.96, 1.6)	0.93 (0.83, 1.0)	1.1 (0.88, 1.3)	1.1 (0.93, 1.3)	1 (0.92, 1.1)	1.1 (0.89, 1.3)	0.93 (0.80, 1.1)	1.1 (0.94, 1.2)	1.1 (1.0, 1.2)	1.2 (0.88, 1.6)	1.1 (0.78, 1.5)	1.1 (0.88, 1.3)	1 (0.81, 1.3)	0.96 (0.79, 1.2)	**Durva**	1 (0.92, 1.1)	1.2 (0.98, 1.4)
1 (0.92, 1.1)	1.2 (0.92, 1.5)	0.89 (0.80,0.99)	1 (0.84, 1.2)	1.1 (0.89, 1.3)	0.97 (0.88, 1.1)	1 (0.85, 1.3)	0.89 (0.76, 1.0)	1 (0.90, 1.2)	1.1 (0.96, 1.2)	1.1 (0.84, 1.5)	1 (0.74, 1.4)	1 (0.84, 1.2)	0.99 (0.77, 1.3)	0.91 (0.75, 1.1)	0.95 (0.87, 1.1)	**Durva+Treme**	1.1 (1.0, 1.3)
1.1 (0.97, 1.4)	1.4 (1.0, 1.8)	1 (0.85, 1.2)	1.2 (0.92, 1.5)	1.2 (0.97, 1.5)	1.1 (0.93, 1.3)	1.2 (0.93, 1.5)	1 (0.83, 1.3)	1.2 (0.96, 1.5)	1.2 (1.0, 1.5)	1.3 (0.93, 1.8)	1.2 (0.83, 1.7)	1.2 (0.92, 1.5)	1.1 (0.85, 1.5)	1.1 (0.82, 1.4)	1.1 (0.92, 1.3)	1.2 (0.99, 1.3)	**Treme**

Data in each cell are hazard ratios for overall survival and progression-free survival with 95% credible intervals in the comparison of the former treatment versus the later treatment. SOC= docetaxel, methotrexate or cetuximab; Durva= Durvalumab; Treme=Tremelimumab.

Figure S2. Network analysis of objective response rate and treatment-related adverse events grade ≥3 adverse events.

Objective response rate (RR, 95%CI) →

Treatment-related adverse events grade >3 (RR. 95%CI) ↓

**SOC**	2.6 (0.73, 13)	0.6 (0.4, 0.88)	5.6 (0.73,160)	1.4 (0.64, 3.2)	0.55 (0.37, 0.79)	0.48 (0.15, 1.3)	1 (0.18, 5.6)	1.7 (0.72, 3.7)	0.42 (0.13, 1.1)	0.53 (0.13, 2.3)	0.73 (0.28, 1.7)	1 (0.12, 9.3)	0.45 (0.02, 5.1)	3.5 (0.87, 18)	1.1 (0.19, 5.8)	0.9 (0.49, 1.6)	0.94 (0.6, 1.4)	0.95 (0.6, 1.5)	6.2 (0.84, 180)
0.8 (0.53, 1.2)	**Cabazitaxel**	0.23 (0.05, 0.87)	2.1 (0.16, 74)	0.52 (0.09, 2.4)	0.21 (0.04, 0.78)	0.18 (0.03, 0.95)	0.37 (0.04, 3.3)	0.62 (0.11, 2.9)	0.15 (0.02, 0.8)	0.2 (0.025, 1.4)	0.27 (0.044, 1.3)	0.38 (0.027, 4.9)	0.16 (0.004, 2.7)	1.3 (0.17, 10)	0.39 (0.04, 3.4)	0.34 (0.064, 1.4)	0.35 (0.07, 1.4)	0.36 (0.071, 1.4)	2.4 (0.18, 83)
2.7 (2.1, 3.6)	3.4 (2.1, 5.5)	**PD-1 inhibitor**	9.4 (1.2, 280)	2.3 (0.97, 5.8)	0.91 (0.52, 1.6)	0.81 (0.24, 2.4)	1.7 (0.29, 9.8)	2.8 (1.1, 6.9)	0.7 (0.21, 2)	0.88 (0.2, 4)	1.2 (0.44, 3.1)	1.7 (0.19, 16)	0.75 (0.024, 8.9)	5.9 (1.4, 31.)	1.8 (0.31, 10)	1.5 (0.73, 3.1)	1.6 (0.86, 2.9)	1.6 (0.87, 2.9)	10 (1.3, 300)
1.5 (0.51, 5)	1.9 (0.6, 6.6)	0.57 (0.18, 1.9)	**Cixutumu-mab+SOC**	0.24 (0.008, 2.2)	0.097 (0.003,0.77)	0.083 (0.003,0.86)	0.17 (0.004, 2.6)	0.29 (0.009, 2.7)	0.071 (0.002,0.73)	0.09 (0.003, 1.2)	0.13 (0.004, 1.2)	0.17 (0.004, 3.7)	0.07 (0.001, 2)	0.61 (0.017, 8.4)	0.18 (0.005, 2.7)	0.16 (0.005,1.3)	0.17 (0.006, 1.3)	0.17 (0.006,1.4)	1.1 (0.024, 53.)
0.82 (0.58, 1.1)	1 (0.61, 1.7)	0.3 (0.2, 0.46)	0.54 (0.16, 1.7)	**Duligotuzu-mab**	0.39 (0.16, 0.92)	0.35 (0.085, 1.2)	0.72 (0.11, 4.8)	1.2 (0.37, 3.7)	0.3 (0.074, 1.0)	0.38 (0.075, 2)	0.52 (0.15, 1.6)	0.74 (0.073, 7.6)	0.32 (0.01, 4.2)	2.5 (0.5, 15.)	0.76 (0.12, 5.)	0.65 (0.23, 1.7)	0.68 (0.27, 1.6)	0.68 (0.27, 1.7)	4.5 (0.51,140)
1 (0.88, 1.2)	1.3 (0.85, 2.0)	0.39 (0.28, 0.53)	0.68 (0.21, 2.0)	1.3 (0.88, 1.8)	**Afatinib**	0.89 (0.26, 2.6)	1.8 (0.32, 11)	3.1 (1.2, 7.5)	0.77 (0.23, 2.2)	0.97 (0.22, 4.4)	1.3 (0.48, 3.4)	1.9 (0.21, 18)	0.82 (0.027, 9.7)	6.4 (1.5, 34)	1.9 (0.34, 11)	1.7 (0.81, 3.4)	1.7 (0.96, 3.1)	1.7 (0.97, 3.1)	12 (1.5, 340)
0.72 (0.53, 0.97)	0.9 (0.55, 1.5)	0.27 (0.18, 0.4)	0.47 (0.14, 1.5)	0.88 (0.56, 1.4)	0.69 (0.49, 0.97)	**PX-866 +SOC**	2.1 (0.29, 16.)	3.5 (0.92, 14)	0.86 (0.19, 3.9)	1.1 (0.19, 6.9)	1.5 (0.38, 6.3)	2.2 (0.20, 25.)	0.93 (0.03, 14)	7.4 (1.3, 52)	2.2 (0.3, 17)	1.9 (0.57, 6.8)	1.9 (0.64, 6.7)	2 (0.65, 6.8)	13 (1.3, 440)
0.9 (0.62, 1.3)	1.1 (0.66, 1.9)	0.33 (0.21, 0.52)	0.59 (0.17, 1.9)	1.1 (0.68, 1.8)	0.87 (0.58, 1.3)	1.2 (0.78, 2.0)	**EMD1201081+SOC**	1.7 (0.24, 11)	0.41 (0.053, 3)	0.53 (0.057, 4.9)	0.72 (0.1, 4.8)	1 (0.065, 16)	0.43 (0.01, 8.8)	3.5 (0.38, 36)	1.1 (0.095,12)	0.89 (0.14, 5.5)	0.93 (0.16, 5.5)	0.95 (0.16, 5.5)	6.6 (0.43, 250)
0.24 (0.09, 0.55)	0.3 (0.098,0.76)	0.09 (0.03, 0.21)	0.16 (0.033,0.63)	0.3 (0.099,0.72)	0.23 (0.081,0.54)	0.34 (0.11, 0.81)	0.27 (0.09, 0.66)	**Zalutumu-mab**	0.25 (0.062,0.91)	0.32 (0.062, 1.7)	0.44 (0.13, 1.4)	0.62 (0.061, 6.5)	0.27 (0.008,3.5)	2.1 (0.42,13)	0.64 (0.098, 4.2)	0.54 (0.2, 1.5)	0.56 (0.22, 1.4)	0.57 (0.23, 1.5)	3.8 (0.43,120)
1.2 (0.84, 1.6)	1.4 (0.87, 2.4)	0.43 (0.28, 0.66)	0.76 (0.22, 2.4)	1.4 (0.90, 2.3)	1.1 (0.78, 1.6)	1.6 (1.0, 2.5)	1.3 (0.8, 2.1)	4.8 (2.0, 14.0)	**Gefitinib +SOC**	1.3 (0.23, 7.9)	1.7 (0.44, 7.2)	2.5 (0.23, 29.)	1.1 (0.031,16.)	8.5 (1.5, 60)	2.6 (0.36, 20)	2.2 (0.68, 7.7)	2.2 (0.77, 7.6)	2.3 (0.77, 7.7)	16 (1.6,500)
0.39 (0.14, 1.0)	0.49 (0.16, 1.4)	0.14 (0.05, 0.39)	0.25 (0.054, 1.1)	0.48 (0.16, 1.3)	0.38 (0.13, 0.98)	0.54 (0.19, 1.5)	0.44 (0.15, 1.2)	1.6 (0.43, 6.6)	0.34 (0.11, 0.91)	**5-FU+SOC**	1.4 (0.32, 5.4)	2 (0.14, 26)	0.83 (0.022, 14)	6.7 (0.88, 57)	2 (0.22, 18.)	1.7 (0.35, 7.9)	1.8 (0.39, 7.8)	1.8 (0.39, 7.9)	12 (0.96, 440)
2.3 (1.7, 3.3)	2.9 (1.7, 4.9)	0.86 (0.56, 1.3)	1.5 (0.45, 4.8)	2.8 (1.8, 4.6)	2.2 (1.5, 3.3)	3.2 (2.1, 5.1)	2.6 (1.6, 4.2)	9.6 (3.9, 29.)	2 (1.3, 3.2)	5.9 (2.3, 17)	**Gefitinib**	1.4 (0.14, 15)	0.62 (0.019, 8.4)	4.9 (0.94, 31.)	1.5 (0.22, 10)	1.2 (0.44, 3.8)	1.3 (0.49, 3.7)	1.3 (0.51, 3.7)	8.9 (0.96, 280)
0.11 (0.03,0.27)	0.14 (0.03,0.37)	0.041 (0.01, 0.11)	0.07 (0.01,0.31)	0.14 (0.03,0.36)	0.11 (0.03,0.27)	0.15 (0.036,0.4)	0.12 (0.03,0.33)	0.45 (0.09, 1.8)	0.095 (0.02,0.25)	0.28 (0.05, 1.1)	0.048 (0.01,0.13)	**Sorafenib +SOC**	0.42 (0.008, 11)	3.4 (0.25, 50)	1 (0.065, 16)	0.87 (0.088, 8.4)	0.91 (0.097, 8.4)	0.92 (0.097, 8.5)	6.4 (0.3,310)
1.1 (0.41, 2.8)	1.3 (0.48, 3.7)	0.4 (0.15, 1.1)	0.7 (0.16, 3)	1.3 (0.48, 3.6)	1 (0.39, 2.7)	1.5 (0.55, 4)	1.2 (0.43, 3.3)	4.4 (1.3, 18.)	0.92 (0.34, 2.5)	2.7 (0.72, 11)	0.46 (0.17, 1.3)	9.9 (2.6, 53)	**Vandetanib +SOC**	8.2 (0.47,330)	2.4 (0.12, 100)	2 (0.16, 63)	2.1 (0.17, 65)	2.1 (0.18, 66)	16 (0.57, 6000)
0.49 (0.24, 0.89)	0.61 (0.27, 1.3)	0.18 (0.083,0.35)	0.32 (0.081, 1.1)	0.6 (0.27, 1.2)	0.47 (0.22, 0.87)	0.68 (0.31, 1.3)	0.55 (0.24, 1.1)	2 (0.67, 6.8)	0.42 (0.19, 0.83)	1.2 (0.38, 4.1)	0.21 (0.1, 0.42)	4.4 (1.4, 21)	0.45 (0.14, 1.4)	**Nolatrexed**	0.3 (0.029, 2.7)	0.26 (0.046, 1.2)	0.27 (0.05, 1.2)	0.27 (0.05, 1.2)	1.8 (0.13, 64)
0.45 (0.23, 0.79)	0.56 (0.26, 1.1)	0.17 (0.08, 0.31)	0.29 (0.076, 1.0)	0.55 (0.26, 1.1)	0.43 (0.22, 0.78)	0.62 (0.3, 1.2)	0.5 (0.23, 0.98)	1.8 (0.64, 6.1)	0.38 (0.18, 0.74)	1.1 (0.36, 3.7)	0.19 (0.091,0.37)	4 (1.3, 19)	0.42 (0.13, 1.3)	0.92 (0.37, 2.3)	**Tivantinib +SOC**	0.85 (0.14, 5.1)	0.89 (0.15, 5.1)	0.9 (0.15, 5.2)	6.2 (0.42, 240)
0.27 (0.13, 0.49)	0.34 (0.15, 0.70)	0.1 (0.046,0.19)	0.18 (0.045,0.62)	0.33 (0.15, 0.66)	0.26 (0.12, 0.48)	0.38 (0.17, 0.74)	0.3 (0.13, 0.61)	1.1 (0.37, 3.8)	0.23 (0.1, 0.46)	0.69 (0.21, 2.3)	0.12 (0.052,0.23)	2.5 (0.76, 11)	0.25 (0.076,0.78)	0.55 (0.22, 1.4)	0.6 (0.24, 1.5)	**Palbociclib+SOC**	1 (0.50, 2.2)	1.1 (0.50, 2.2)	7 (0.85, 210)
2.3 (1.5, 3.6)	2.9 (1.6, 5.2)	0.86 (0.51, 1.4)	1.5 (0.43, 4.9)	2.8 (1.6, 4.9)	2.2 (1.4, 3.6)	3.2 (1.9, 5.5)	2.6 (1.5, 4.6)	9.5 (3.8, 30.)	2 (1.2, 3.4)	5.9 (2.1, 18.)	0.99 (0.57, 1.7)	21 (7.6, 92.)	2.2 (0.76, 6.1)	4.7 (2.2, 11)	5.2 (2.5, 11)	8.5 (4.0, 20)	**Durva**	1 (0.57, 1.8)	6.7 (0.86, 190)
1.5 (1.0, 2.3)	1.9 (1.1, 3.3)	0.57 (0.35, 0.91)	1 (0.29, 3.2)	1.9 (1.1, 3.1)	1.5 (0.96, 2.2)	2.1 (1.3, 3.5)	1.7 (1.0, 2.9)	6.3 (2.5, 19.)	1.3 (0.79, 2.2)	3.9 (1.4, 12.)	0.66 (0.39, 1.1)	14 (5.1, 61)	1.4 (0.51, 4)	3.1 (1.5, 7.1)	3.4 (1.7, 7.4)	5.6 (2.7, 13)	0.66 (0.39, 1.1)	**Durva +Treme**	6.5 (0.93, 180)
1.4 (0.55, 3.3)	1.7 (0.64, 4.5)	0.51 (0.20, 1.3)	0.89 (0.20, 3.7)	1.7 (0.64, 4.3)	1.3 (0.52, 3.3)	1.9 (0.73, 4.9)	1.5 (0.58, 4)	5.7 (1.7, 22.)	1.2 (0.45, 3.0)	3.5 (0.95, 14)	0.59 (0.22, 1.5)	13 (3.4, 65.)	1.3 (0.35, 4.7)	2.8 (0.94, 8.8)	3.1 (1.0, 9.3)	5.1 (1.7, 16.)	0.59 (0.23, 1.5)	0.9 (0.39,2)	**Treme**

Data in each cell are risk ratios for objective response rate and grade ≥3 adverse events with 95% credible intervals in the comparison of the former treatment versus the later treatment. SOC= docetaxel, methotrexate or cetuximab; Durva= Durvalumab; Treme=Tremelimumab.

Figure S3. Rank probabilities of overall survival (A), progression free survival (B), objective response rate (C) and adverse events of grade 3 or higher (D).

A

Rank Probabilities of OS	[,1]	[,2]	[,3]	[,4]	[,5]	[,6]	[,7]	[,8]	[,9]	[,10]	[,11]	[,12]	[,13]	[,14]	[,15]	[,16]	[,17]	[,18]
SOC	0%	0%	0%	0%	0%	2%	6%	14%	22%	24%	18%	9%	4%	1%	0%	0%	0%	0%
Cabazitaxel	0%	0%	1%	1%	1%	1%	1%	1%	1%	2%	2%	3%	4%	5%	7%	11%	21%	38%
PD-1 inhibitor	11%	23%	26%	20%	11%	6%	3%	1%	0%	0%	0%	0%	0%	0%	0%	0%	0%	0%
Cixutumumab+SOC	2%	3%	3%	4%	5%	5%	5%	5%	5%	5%	6%	7%	8%	8%	9%	9%	7%	4%
Duligotuzumab	0%	1%	1%	2%	2%	3%	4%	4%	4%	5%	6%	8%	9%	11%	12%	13%	11%	5%
Afatinib	0%	1%	2%	5%	10%	14%	16%	15%	12%	9%	7%	5%	3%	1%	1%	0%	0%	0%
EMD1201081+SOC	1%	2%	3%	3%	4%	5%	5%	5%	5%	5%	6%	7%	8%	9%	10%	10%	9%	5%
Zalutumumab	18%	20%	17%	14%	10%	7%	4%	3%	2%	2%	1%	1%	1%	0%	0%	0%	0%	0%
Gefitinib+SOC	0%	0%	1%	2%	3%	4%	5%	6%	6%	8%	9%	11%	12%	11%	10%	7%	4%	1%
Vandetanib+SOC	8%	5%	5%	4%	4%	4%	4%	3%	3%	3%	3%	4%	5%	5%	6%	9%	11%	13%
Nolatrexed	1%	2%	4%	5%	6%	6%	6%	6%	6%	6%	7%	8%	8%	8%	8%	7%	5%	2%
Gefitinib	0%	0%	0%	0%	0%	0%	1%	1%	2%	4%	6%	11%	15%	19%	19%	14%	7%	1%
Sorafenib+SOC	2%	2%	2%	2%	2%	2%	2%	2%	2%	2%	3%	4%	4%	5%	7%	10%	18%	28%
Tivantinib+SOC	7%	6%	6%	6%	6%	6%	5%	5%	4%	4%	5%	5%	6%	6%	6%	7%	6%	4%
Palbociclib+SOC	16%	14%	12%	11%	9%	7%	6%	5%	4%	3%	3%	3%	3%	2%	2%	1%	1%	0%
Durvalumab	0%	2%	5%	10%	15%	17%	15%	11%	8%	6%	4%	3%	2%	1%	0%	0%	0%	0%
Durva+Treme	0%	0%	0%	1%	3%	6%	9%	11%	12%	12%	13%	12%	9%	6%	3%	1%	0%	0%
Tremelimumab	34%	19%	13%	10%	7%	5%	3%	2%	2%	1%	1%	1%	1%	1%	0%	0%	0%	0%

B

Rank Probabilities of PFS	[,1]	[,2]	[,3]	[,4]	[,5]	[,6]	[,7]	[,8]	[,9]	[,10]	[,11]	[,12]	[,13]	[,14]	[,15]	[,16]	[,17]
SOC	0%	0%	0%	0%	0%	3%	10%	19%	25%	22%	13%	6%	2%	0%	0%	0%	0%
Cabazitaxel	0%	0%	0%	1%	3%	5%	7%	7%	7%	8%	9%	10%	11%	11%	10%	7%	3%
PD-1 inhibitor	0%	0%	1%	4%	12%	20%	21%	16%	11%	7%	5%	3%	1%	1%	0%	0%	0%
Cixutumumab+SOC	44%	24%	13%	8%	5%	3%	1%	1%	1%	0%	0%	0%	0%	0%	0%	0%	0%
Duligotuzumab	0%	0%	0%	0%	1%	2%	2%	3%	3%	4%	5%	7%	10%	13%	16%	18%	16%
Afatinib	1%	9%	26%	32%	21%	8%	2%	1%	0%	0%	0%	0%	0%	0%	0%	0%	0%
EMD1201081+SOC	0%	1%	2%	3%	5%	6%	6%	5%	5%	5%	6%	7%	8%	9%	10%	11%	10%
Zalutumumab	28%	37%	20%	9%	4%	2%	1%	0%	0%	0%	0%	0%	0%	0%	0%	0%	0%
Gefitinib+SOC	0%	0%	0%	0%	1%	2%	2%	3%	3%	4%	5%	7%	10%	13%	16%	19%	17%
Vandetanib+SOC	15%	9%	7%	6%	6%	5%	4%	3%	2%	2%	3%	3%	3%	4%	5%	6%	17%
Nolatrexed	0%	1%	1%	3%	5%	7%	7%	7%	6%	6%	7%	8%	9%	9%	9%	8%	5%
Sorafenib+SOC	3%	4%	6%	6%	8%	7%	6%	5%	4%	4%	4%	5%	6%	6%	7%	8%	11%
Tivantinib+SOC	0%	1%	1%	2%	4%	5%	5%	4%	4%	5%	5%	6%	8%	9%	11%	13%	15%
Palbociclib+SOC	1%	2%	4%	6%	9%	10%	9%	7%	6%	6%	6%	7%	7%	7%	6%	5%	4%
Durvalumab	0%	0%	0%	0%	1%	3%	5%	8%	10%	12%	15%	15%	13%	9%	5%	2%	1%
Durva+Treme	0%	0%	0%	0%	1%	4%	7%	9%	11%	13%	15%	14%	11%	8%	4%	2%	0%
Tremelimumab	7%	14%	19%	18%	15%	10%	6%	3%	2%	2%	1%	1%	1%	0%	0%	0%	0%

C

Rank Probabilities of ORR	[,1]	[,2]	[,3]	[,4]	[,5]	[,6]	[,7]	[,8]	[,9]	[,10]	[,11]	[,12]	[,13]	[,14]	[,15]	[,16]	[,17]	[,18]	[,19]	[,20]
SOC	0%	0%	0%	0%	0%	0%	1%	5%	12%	20%	24%	20%	12%	5%	1%	0%	0%	0%	0%	0%
Cabazitaxel	0%	0%	0%	0%	0%	1%	1%	1%	1%	1%	2%	2%	4%	6%	9%	13%	18%	19%	15%	7%
PD-1 inhibitor	1%	4%	10%	15%	18%	18%	15%	10%	5%	2%	1%	0%	0%	0%	0%	0%	0%	0%	0%	0%
Cixutumumab+SOC	0%	0%	0%	0%	0%	0%	1%	1%	1%	1%	1%	1%	2%	2%	4%	6%	9%	13%	23%	35%
Duligotuzumab	0%	0%	0%	0%	1%	1%	2%	3%	4%	5%	6%	8%	11%	15%	16%	13%	8%	4%	1%	0%
Afatinib	2%	7%	15%	20%	20%	16%	11%	6%	2%	1%	0%	0%	0%	0%	0%	0%	0%	0%	0%	0%
PX-866+SOC	13%	17%	15%	11%	9%	8%	6%	5%	4%	3%	2%	2%	2%	1%	1%	0%	0%	0%	0%	0%
EMD1201081+SOC	5%	6%	5%	5%	5%	5%	5%	5%	4%	4%	4%	5%	6%	7%	8%	8%	7%	5%	3%	1%
Zalutumumab	0%	0%	0%	0%	0%	1%	1%	2%	2%	3%	3%	5%	9%	13%	18%	19%	14%	7%	2%	0%
Gefitinib+SOC	18%	21%	16%	11%	8%	6%	5%	4%	3%	2%	1%	1%	1%	1%	0%	0%	0%	0%	0%	0%
Vandetanib+SOC	33%	10%	7%	5%	4%	3%	3%	3%	3%	2%	2%	2%	3%	3%	4%	3%	3%	3%	2%	1%
Nolatrexed	0%	0%	0%	0%	0%	0%	0%	1%	1%	1%	1%	1%	2%	4%	6%	9%	16%	23%	23%	12%
5-FU+SOC	14%	15%	12%	9%	7%	6%	6%	5%	4%	3%	3%	3%	3%	3%	3%	2%	1%	1%	0%	0%
Gefitinib	2%	5%	7%	8%	9%	10%	10%	9%	8%	6%	6%	5%	5%	4%	3%	1%	1%	0%	0%	0%
Sorafenib+SOC	8%	7%	5%	4%	4%	4%	4%	4%	4%	3%	3%	4%	5%	6%	7%	7%	7%	6%	5%	3%
Tivantinib+SOC	4%	5%	5%	5%	4%	4%	5%	5%	4%	4%	4%	5%	6%	7%	8%	8%	7%	5%	3%	1%
Palbociclib+SOC	0%	1%	1%	3%	4%	7%	9%	11%	12%	11%	10%	9%	9%	7%	4%	2%	1%	0%	0%	0%
Durvalumab	0%	0%	0%	1%	2%	4%	8%	11%	13%	14%	13%	12%	10%	6%	3%	1%	0%	0%	0%	0%
Durva+Treme	0%	0%	0%	1%	2%	4%	7%	11%	13%	14%	13%	12%	10%	7%	3%	1%	0%	0%	0%	0%
Tremelimumab	0%	0%	0%	0%	0%	0%	0%	0%	1%	1%	1%	1%	1%	2%	3%	5%	8%	13%	23%	39%

D

Rank Probabilities of AEs	[,1]	[,2]	[,3]	[,4]	[,5]	[,6]	[,7]	[,8]	[,9]	[,10]	[,11]	[,12]	[,13]	[,14]	[,15]	[,16]	[,17]	[,18]	[,19]	[,20]
SOC	0%	0%	0%	0%	0%	0%	0%	0%	2%	10%	26%	32%	20%	7%	1%	0%	0%	0%	0%	0%
Cabazitaxel	0%	0%	0%	0%	2%	7%	15%	19%	19%	15%	9%	6%	4%	3%	1%	0%	0%	0%	0%	0%
PD-1 inhibitor	0%	0%	0%	0%	0%	0%	0%	0%	0%	0%	0%	0%	0%	0%	0%	1%	4%	15%	33%	46%
Cixutumumab+SOC	0%	0%	0%	1%	1%	2%	3%	3%	3%	4%	3%	3%	4%	7%	11%	12%	14%	8%	6%	13%
Duligotuzumab	0%	0%	0%	0%	1%	4%	13%	19%	22%	18%	10%	6%	4%	2%	1%	0%	0%	0%	0%	0%
Afatinib	0%	0%	0%	0%	0%	0%	0%	1%	3%	8%	15%	22%	26%	18%	7%	2%	0%	0%	0%	0%
PX-866+SOC	0%	0%	0%	0%	3%	13%	29%	25%	16%	9%	4%	1%	0%	0%	0%	0%	0%	0%	0%	0%
EMD1201081+SOC	0%	0%	0%	0%	0%	2%	7%	11%	16%	18%	14%	10%	9%	7%	3%	1%	0%	0%	0%	0%
Zalutumumab	12%	38%	25%	14%	7%	3%	1%	0%	0%	0%	0%	0%	0%	0%	0%	0%	0%	0%	0%	0%
Gefitinib+SOC	0%	0%	0%	0%	0%	0%	0%	1%	2%	4%	6%	9%	17%	26%	21%	11%	3%	0%	0%	0%
Vandetanib+SOC	0%	0%	1%	1%	3%	5%	7%	6%	7%	7%	6%	5%	7%	11%	11%	10%	7%	3%	2%	2%
Nolatrexed	0%	3%	7%	17%	26%	25%	10%	5%	3%	2%	1%	0%	0%	0%	0%	0%	0%	0%	0%	0%
5-FU+SOC	2%	12%	17%	22%	18%	13%	5%	3%	2%	1%	1%	1%	1%	1%	0%	0%	0%	0%	0%	0%
Gefitinib	0%	0%	0%	0%	0%	0%	0%	0%	0%	0%	0%	0%	0%	0%	1%	5%	17%	33%	29%	15%
Sorafenib+SOC	81%	14%	4%	1%	0%	0%	0%	0%	0%	0%	0%	0%	0%	0%	0%	0%	0%	0%	0%	0%
Tivantinib+SOC	0%	3%	10%	23%	30%	22%	7%	3%	2%	1%	0%	0%	0%	0%	0%	0%	0%	0%	0%	0%
Palbociclib+SOC	4%	30%	36%	19%	7%	2%	0%	0%	0%	0%	0%	0%	0%	0%	0%	0%	0%	0%	0%	0%
Durvalumab	0%	0%	0%	0%	0%	0%	0%	0%	0%	0%	0%	0%	0%	0%	2%	6%	18%	30%	24%	19%
Durva+Treme	0%	0%	0%	0%	0%	0%	0%	0%	0%	0%	1%	1%	2%	8%	24%	36%	23%	4%	1%	0%
Tremelimumab	0%	0%	0%	0%	1%	2%	3%	3%	4%	4%	4%	4%	5%	9%	15%	16%	12%	6%	4%	5%

Figure S4. Pairwise meta-analyses in overall survival of afatinib vs SOC and PD-1 inhibitor vs SOC.

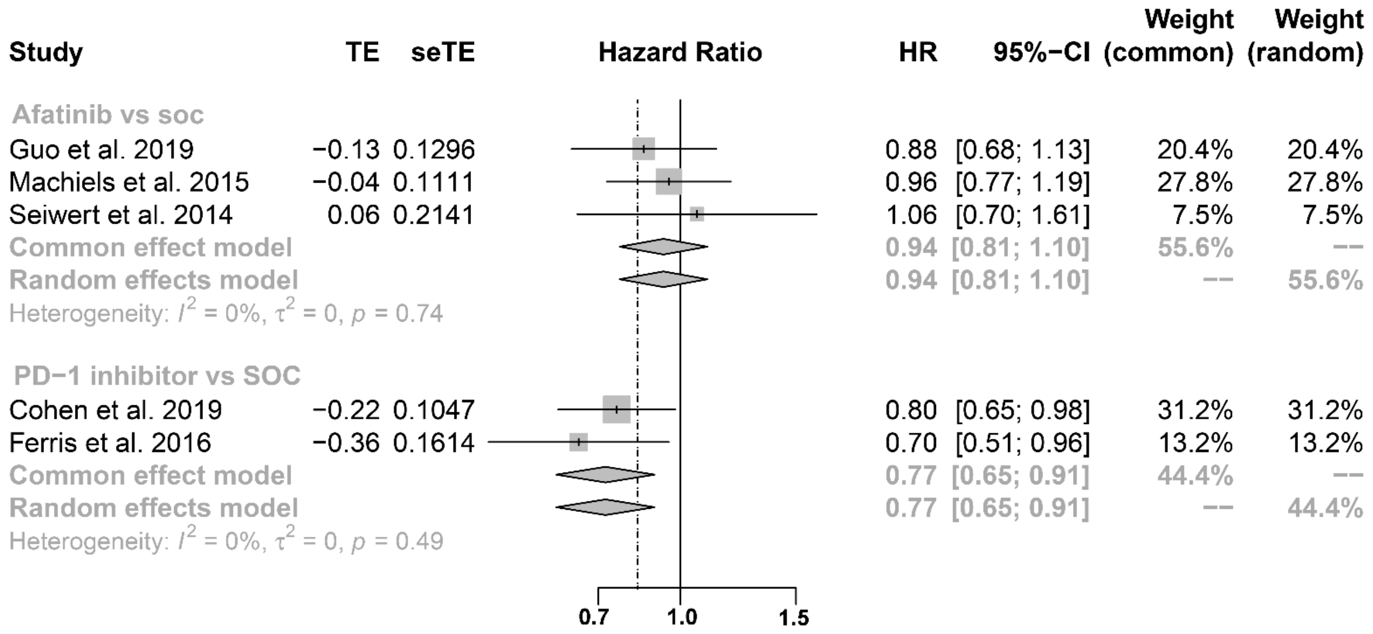

Figure S5. Pairwise meta-analyses in progression free survival of afatinib vs SOC, PD-1 inhibitor vs SOC and cabazitaxel vs SOC.

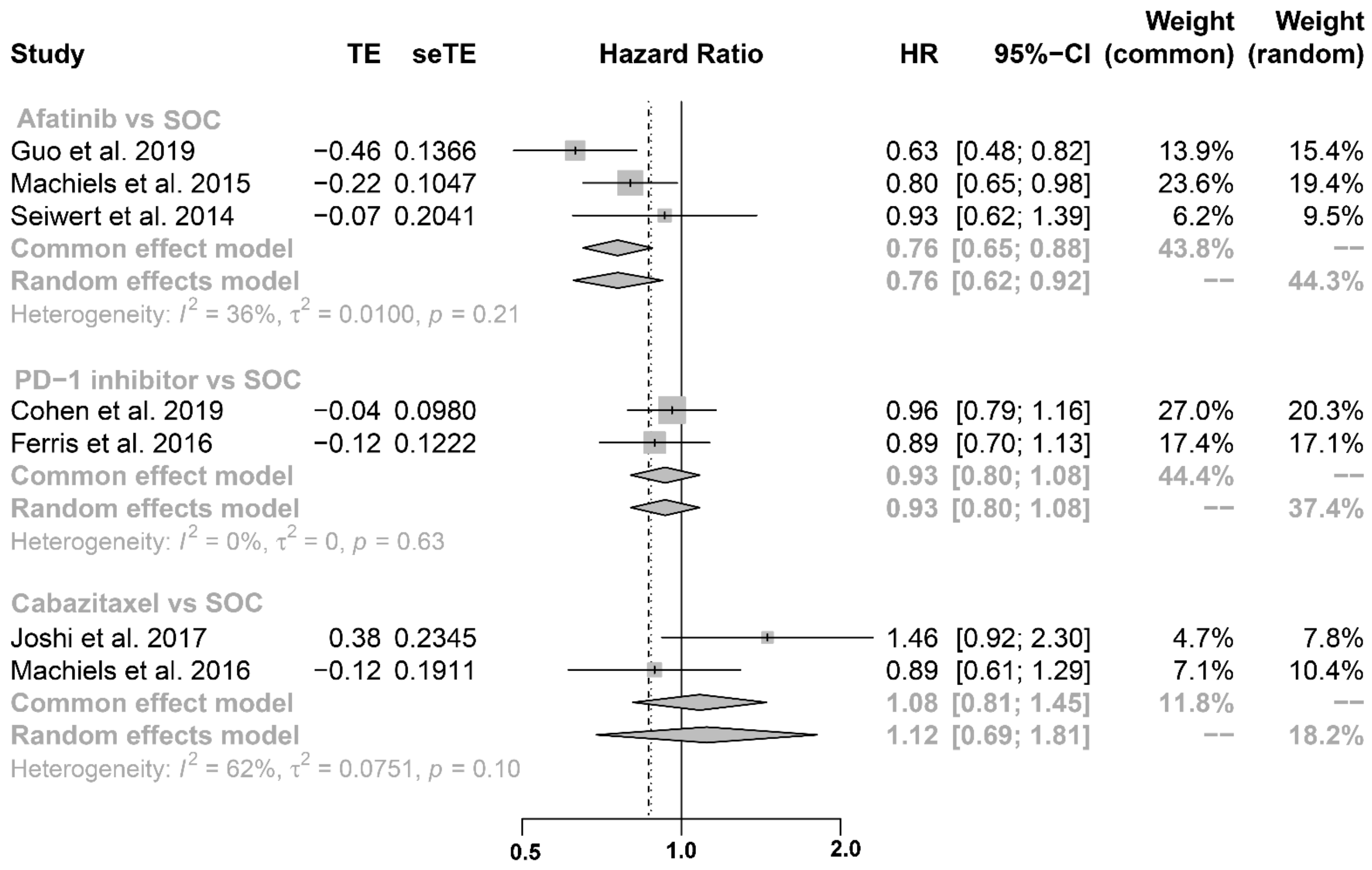

Figure S6. Pairwise meta-analyses in objective response rate of afatinib vs SOC, PD-1 inhibitor vs SOC, PX-866+SOC vs SOC, and gefitinib vs SOC.

Study	Arm_1 Events	Arm_1 Total	Arm_2 Events	Arm_2 Total	RR	95%−CI	Weight (common)	Weight (random)
**Afatinib vs SOC**								
Guo et al. 2019	64	228	15	112	2.10	[1.25; 3.51]	23.5%	26.2%
Machiels et al. 2015	33	322	9	161	1.83	[0.90; 3.74]	14.0%	13.7%
Seiwert et al. 2014	5	62	6	62	0.83	[0.27; 2.59]	7.0%	5.4%
Common effect model		612		335	1.81	[1.23; 2.68]	44.6%	--
Random effects model					1.80	[1.22; 2.67]	--	45.4%
Heterogeneity: $I^2 = 5\%$, $\tau^2 = < 0.0001$, $p = 0.35$								
**PD−1 inhibitor vs SOC**								
Cohen et al. 2019	36	247	25	248	1.45	[0.90; 2.33]	29.2%	30.3%
Ferris et al. 2016	32	240	7	121	2.30	[1.05; 5.07]	10.9%	11.2%
Common effect model		487		369	1.68	[1.11; 2.53]	40.1%	--
Random effects model					1.64	[1.09; 2.47]	--	41.5%
Heterogeneity: $I^2 = 0\%$, $\tau^2 = 0$, $p = 0.32$								
**PX−866+SOC vs SOC**								
Jimeno et al. 2015	4	42	3	41	1.30	[0.31; 5.46]	3.6%	3.4%
Jimeno et al. 2015	6	42	2	43	3.07	[0.66; 14.37]	2.3%	2.9%
Common effect model		84		84	2.00	[0.71; 5.60]	5.9%	--
Random effects model					1.94	[0.68; 5.54]	--	6.3%
Heterogeneity: $I^2 = 0\%$, $\tau^2 = 0$, $p = 0.42$								
**Gefitinib vs SOC**								
Kushwaha et al. 2015	3	39	2	40	1.54	[0.27; 8.71]	2.3%	2.3%
Stewart et al. 2009	4	158	6	152	0.64	[0.18; 2.23]	7.2%	4.5%
Common effect model		197		192	0.86	[0.32; 2.32]	9.5%	--
Random effects model					0.86	[0.31; 2.38]	--	6.8%
Heterogeneity: $I^2 = 0\%$, $\tau^2 = 0$, $p = 0.42$								

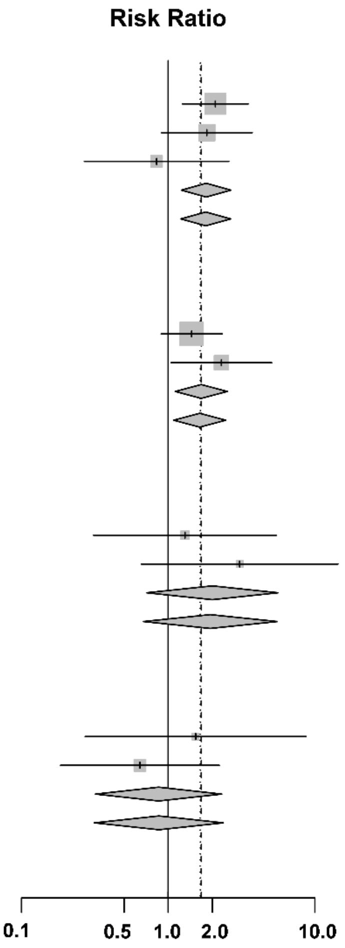

Figure S7. Pairwise meta-analyses in adverse events of grade 3 or higher of afatinib vs SOC, PD-1 inhibitor vs SOC, Cabazitaxel vs SOC, PX-866+SOC vs SOC and gefitinib vs SOC.

Study	Arm_1 Events	Arm_1 Total	Arm_2 Events	Arm_2 Total	RR	95%−CI	Weight (common)	Weight (random)
**Afatinib vs SOC**								
Guo et al. 2019	89	228	67	112	0.65	[0.52; 0.81]	20.3%	10.4%
Machiels et al. 2015	127	320	57	160	1.11	[0.87; 1.43]	17.1%	10.3%
Seiwert et al. 2014	32	62	11	62	2.91	[1.62; 5.24]	2.5%	9.0%
Common effect model		610		334	0.99	[0.85; 1.16]	39.9%	--
Random effects model					1.24	[0.54; 2.83]	--	29.8%
Heterogeneity: $I^2 = 92\%$, $\tau^2 = 0.4974$, $p < 0.01$								
**PD−1 inhibitor vs SOC**								
Cohen et al. 2019	33	246	85	234	0.37	[0.26; 0.53]	19.6%	10.0%
Ferris et al. 2016	31	236	39	111	0.37	[0.25; 0.57]	12.0%	9.8%
Common effect model		482		345	0.37	[0.28; 0.49]	31.6%	--
Random effects model					0.37	[0.28; 0.49]	--	19.8%
Heterogeneity: $I^2 = 0\%$, $\tau^2 = 0$, $p = 0.97$								
**PX−866+SOC vs SOC**								
Jimeno et al. 2015	25	40	21	39	1.16	[0.80; 1.69]	4.8%	9.9%
Jimeno et al. 2015	26	42	15	43	1.77	[1.11; 2.85]	3.3%	9.6%
Common effect model		82		82	1.41	[1.05; 1.90]	8.1%	--
Random effects model					1.40	[0.93; 2.12]	--	19.5%
Heterogeneity: $I^2 = 47\%$, $\tau^2 = 0.0426$, $p = 0.17$								
**Gefitinib vs SOC**								
Kushwaha et al. 2015	1	39	5	40	0.21	[0.03; 1.68]	1.1%	3.2%
Stewart et al. 2009	16	158	56	159	0.29	[0.17; 0.48]	12.6%	9.4%
Common effect model		197		199	0.28	[0.17; 0.46]	13.7%	--
Random effects model					0.28	[0.17; 0.46]	--	12.6%
Heterogeneity: $I^2 = 0\%$, $\tau^2 = 0$, $p = 0.76$								
**Cabazitaxel vs SOC**								
Joshi et al. 2017	17	46	16	46	1.06	[0.61; 1.84]	3.6%	9.2%
Machiels et al. 2016	21	53	13	48	1.46	[0.83; 2.59]	3.1%	9.1%
Common effect model		99		94	1.25	[0.84; 1.85]	6.7%	--
Random effects model					1.24	[0.83; 1.84]	--	18.3%
Heterogeneity: $I^2 = 0\%$, $\tau^2 = 0$, $p = 0.43$								

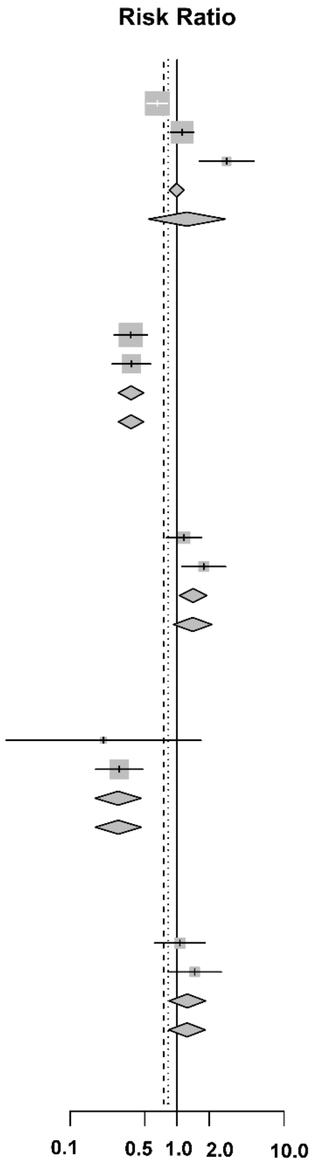

Figure S8. Sensitivity analysis in the phase III trials.

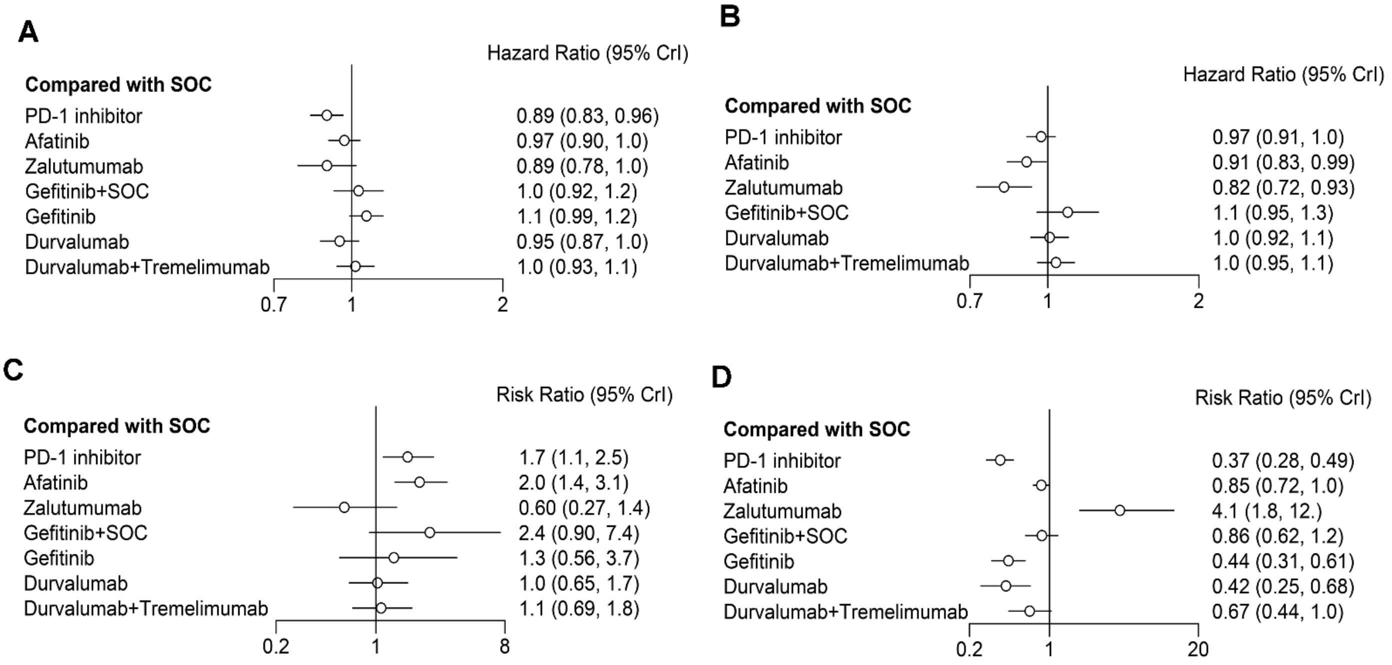

Figure S9. Sensitivity analysis of methotrexate.

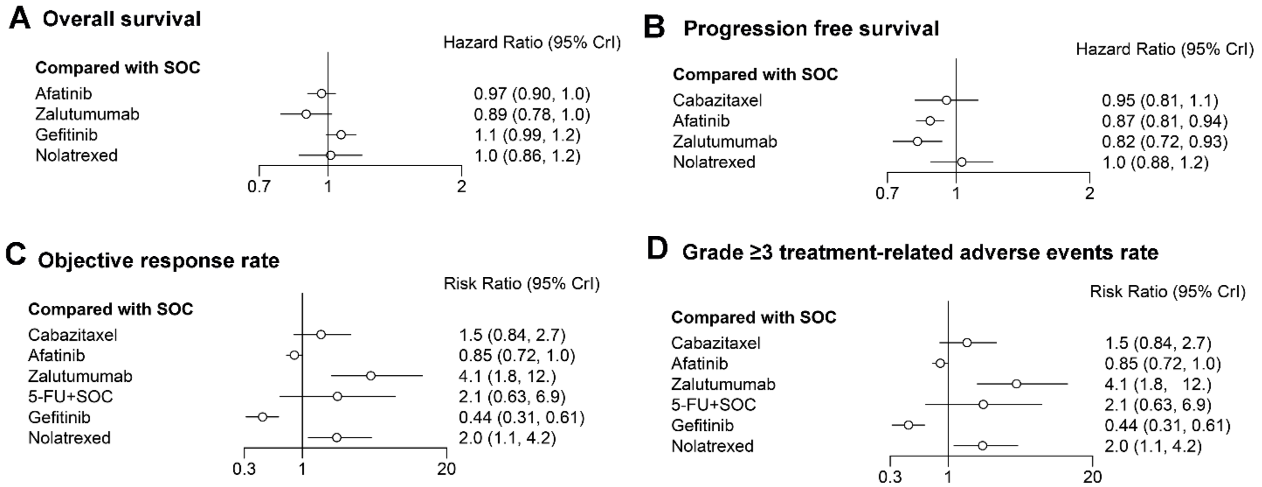

Figure S10. Sensitivity analysis of cetuximab.

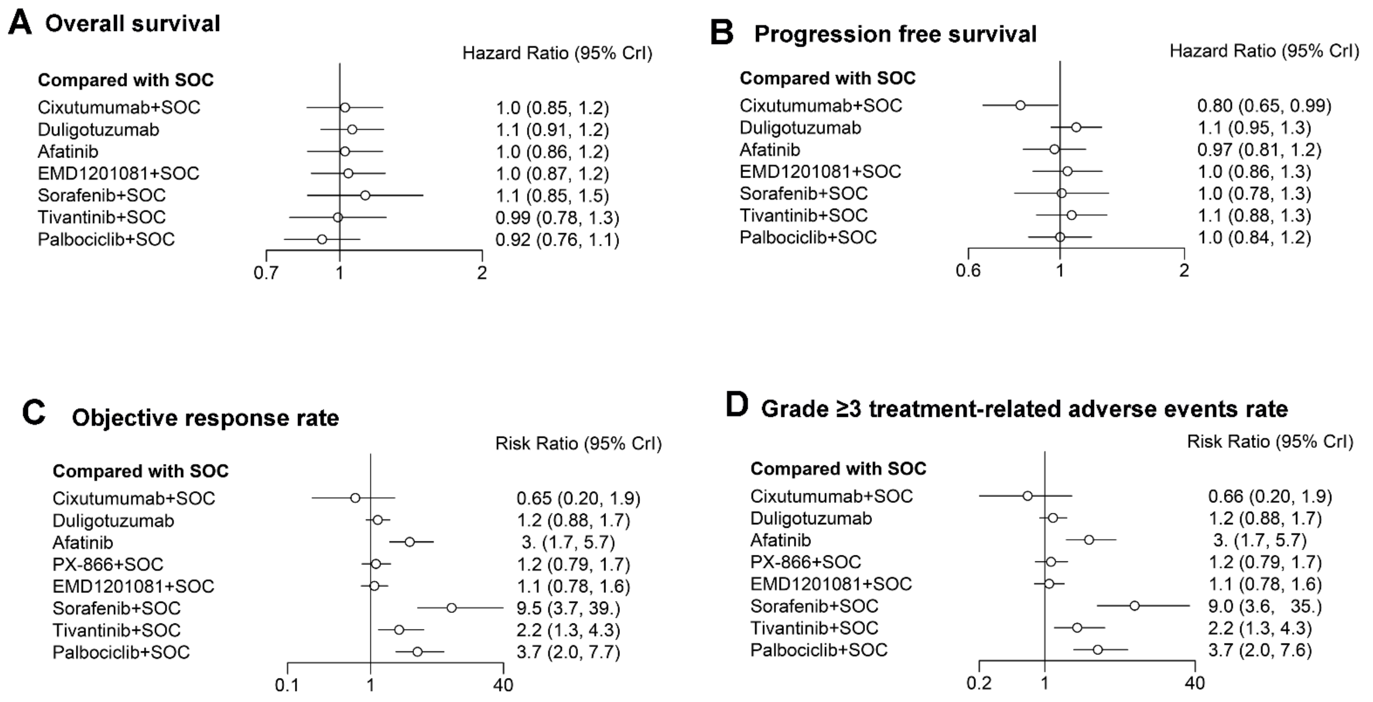

Figure S11. Sensitivity analysis of docetaxel.

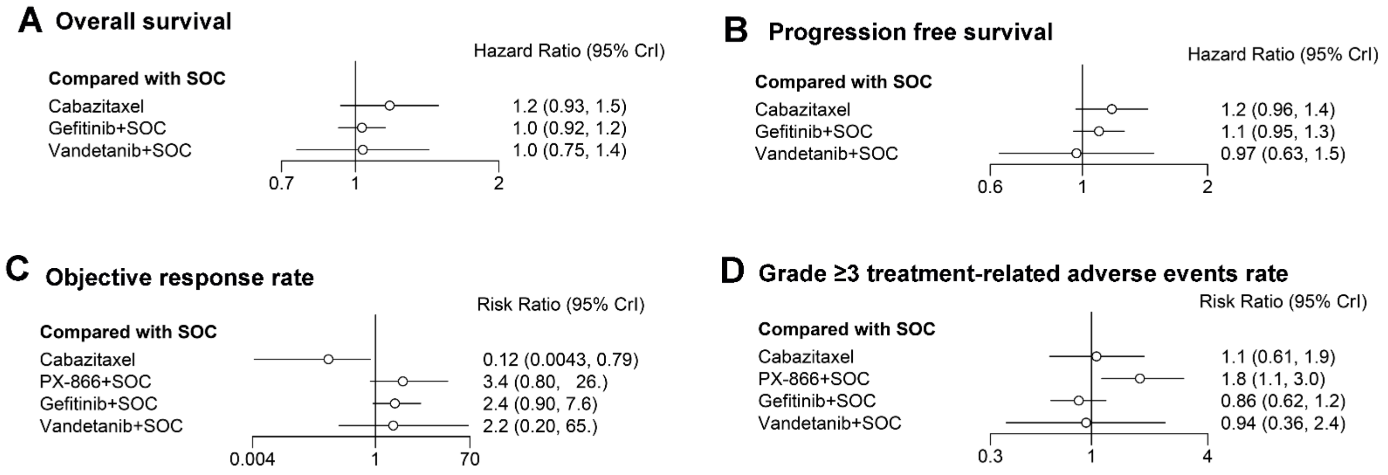

Supplement: Supplementary file 1 [file cancers-14-04472-s001.zip › cancers-1866899-supplementary.pdf]
